# Real-Hardware Deployment of a Nussbaum-Function PID Controller on a Current-Controlled Low-Cost Actuator via Hardware-Aware Optuna Tuning

**DOI:** 10.3390/s26134212

**Published:** 2026-07-03

**Authors:** Danial Zafaranchizadeh Moghaddam, Olga Tveretina, Abolfazl Zaraki

**Affiliations:** School of Physics, Engineering and Computer Science (SPECS), University of Hertfordshire, Hatfield AL10 9AB, UK; dz24aaf@herts.ac.uk (D.Z.M.); o.tveretina@herts.ac.uk (O.T.)

**Keywords:** robot manipulator control, Nussbaum function, adaptive PID, real-hardware validation, Dynamixel current control, Optuna, low-cost robotics, trajectory tracking, actuator saturation

## Abstract

Real-hardware deployment of adaptive manipulator controllers remains difficult because assumptions made in paper-level formulations are weakened by friction, encoder quantisation, current limits, communication latency, and low-speed actuation nonlinearities. This paper investigates that deployment gap for a recent Nussbaum-function PID controller by translating it from a simulation-level formulation into direct current-command control on a Niryo NED3 Pro actuator. To isolate deployment-layer behaviour from the whole-arm Coriolis, centrifugal, and gravity dynamics, the study is centred on a single decoupled actuator (Dynamixel ID 6, distal wrist) under long-horizon sinusoidal tracking around a fixed operating region. A direct transfer of the baseline law is first reproduced and shown to degrade through cumulative adaptation-state growth, weakening of the Nussbaum modulation, and high internal command saturation. A hardware-oriented implementation is then evaluated that preserves the Nussbaum core while adding adaptation-state regularisation, low-speed velocity-reference feedforward, and tail-region damping; its parameters are selected through a hardware-aware Optuna archive of 79 real-hardware trials with hard rejection of unsafe runs and a score that jointly reflects tracking quality, internal command saturation, actuation activity, and bounded adaptation growth. Over 300 s of continuous operation, the enhanced implementation reduces the mean absolute error from $10.476^{\circ }$ to $1.054^{\circ }$ and the internal command saturation ratio from 0.450 to 0.012 relative to the direct baseline, within the reported actuator, trajectory, and safety envelope. The main contribution is therefore not only a tuned controller but a reproducible real-hardware methodology showing how Nussbaum-based PID control can be deployed and improved on a low-cost manipulator when adaptation management, actuation mapping, and hardware-aware optimisation are treated as core elements of the research design.

## 1. Introduction

**Figure 1:**
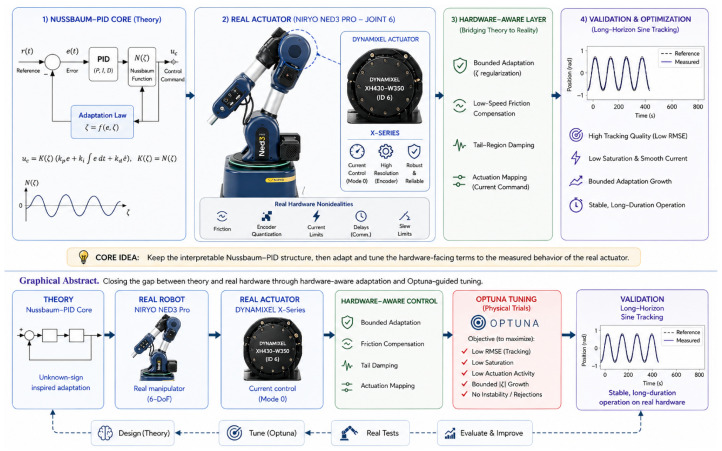
Conceptual graphical abstract of the study: Nussbaum-PID core is connected to a real current-driven actuator, augmented by hardware-aware regularisation, and tuned through measured physical trials before long-horizon validation. The project landing page is hosted at https://danielz.co.uk/projects/hardware-aware-nussbaum-pid/ (accessed on 26 June 2026), and the companion GitHub repository (manuscript source, figures, tables, and scripts; accepted-manuscript archive version, 25 June 2026) is available at https://github.com/danialza/hardware-aware-nussbaum-pid (accessed on 26 June 2026).

Reliable trajectory tracking on low-cost robot manipulators is still much harder in real hardware than in simulation. Even when a controller is supported by Lyapunov analysis, its practical behaviour can be dominated by effects that are usually suppressed or idealised in paper-level models, including friction, gear compliance, encoder quantisation, actuator current limits, communication delay, and command slew limits. Recent reviews continue to show that adaptive, robust, disturbance-observer, and neural techniques remain central to manipulator control, especially when uncertainties and constraints matter [1,2]. However, the literature also shows a persistent deployment gap: many methods are evaluated numerically or on highly controlled laboratory platforms, while the transition to low-cost current-driven actuators remains underreported. A central part of this gap is sensing: on low-cost actuators, the controller closes its loop on quantised position feedback and on a noisy, current-limited torque-proxy measurement, so the practical behaviour of an adaptive law is inseparable from the resolution, latency, and saturation of the on-board sensing and current-measurement chain. The present study therefore treats encoder quantisation, velocity estimation, and current feedback not as nuisance terms but as first-class measurement constraints that govern whether the adaptive law remains usable. Figure 1 provides a compact visual overview of this deployment pipeline: a published Nussbaum-PID core is connected to a real current-driven actuator, augmented by hardware-aware regularisation, and tuned using measured physical trials before the final validation runs.

In the present project, that gap became explicit when a recent Nussbaum-function PID formulation for robot manipulators [3] was moved from simulation-level validation to direct current-command control on a Niryo NED3 Pro wrist actuator (Niryo, Wambrechies, France). The appeal of the Nussbaum approach is clear. Since the early Nussbaum-gain idea for adaptive systems with unknown control direction [4], this class of methods has remained attractive because it can handle sign-sensitive control effectiveness without requiring the sign of the high-frequency gain to be known a priori. In robot and manipulator control contexts, this means that a compact adaptive controller can be designed even when the effective actuator direction, transmission convention, or low-level command mapping is uncertain [5,6]. Yet the same feature that makes Nussbaum methods attractive in theory, namely the oscillatory adaptive modulation generated by N(ζ), can become practically fragile if the adaptation state ζ is allowed to drift through critical regions while the actuator is already current-limited or friction-dominated.

This paper therefore has two linked goals. First, it documents a faithful real-hardware implementation of the baseline Nussbaum-PID (NPID) formulation on a directly commanded Dynamixel actuator. Second, it evaluates a hardware-oriented enhanced NPID implementation that preserves the Nussbaum core while adding implementation-level regularisation required for long-duration operation on the tested real motor. To make this deployment behaviour observable under controlled conditions, the evaluation is deliberately concentrated on a single distal-wrist actuator, so that dominant whole-arm Coriolis, centrifugal, and gravity coupling effects are reduced and the measured residual is more strongly governed by the adaptation mechanism and the actuator/sensing chain; the rationale for this controlled single-joint design is detailed in Section 3. The resulting study is intentionally not framed as a pure theoretical extension. Instead, it is framed as a real-hardware control paper in which implementation-layer decisions are treated as research contributions because they determine whether the adaptive law remains usable beyond short favourable windows.

This distinction is important for the novelty claim. The individual engineering elements added in the enhanced implementation, such as leakage, feedforward, damping, anti-windup, and saturation guards, are not claimed here as new control-theoretic mechanisms in isolation. The contribution is the experimentally documented deployment pathway: translating the NPID law into a direct current-command actuator layer, showing the real-hardware degradation mechanism caused by uncontrolled ζ-growth under current-limited operation, and demonstrating that a hardware-aware combination of adaptation management, command mapping, and Optuna-based physical tuning can recover sustained operation under the stated actuator envelope.

A second contribution of the paper is methodological. Instead of relying on manual parameter tuning alone, the controller was tuned through an aggregated Optuna archive on the physical actuator. Across the archived bundle, this search accumulated 79 real-hardware trials and approximately 87.9 min of closed-loop runtime. Optuna was selected because it supports structured trial management, dynamic search spaces, and efficient search through tree-structured Parzen estimators [7]. More importantly for this study, the objective was defined in hardware terms rather than as pure curve-fitting: unsafe or practically poor behaviours such as insufficient motion span, high internal command saturation, excessive ζ-growth, and large long-tail errors were rejected or penalised directly in the scoring function. This turned the tuning process itself into a reproducible part of the research method.

The main contributions of the paper are as follows:

- A direct current-command implementation of a recent Nussbaum-function PID on a low-cost real manipulator actuator, with full run logging and reproducible plots.
- A hardware-oriented enhanced NPID implementation that retains the Nussbaum core but adds soft ζ-leakage, velocity-reference feedforward, and tail-region damping to reduce deployment-induced degradation on the tested actuator without claiming those individual engineering terms as stand-alone theoretical innovations.
- An Optuna-based real-hardware tuning archive with 79 physical trials, hard safety filters, and multi-objective score shaping focused on tracking accuracy, saturation, actuation activity, and bounded adaptation growth.
- A 300 s decoupled-joint experimental dataset, obtained on the Dynamixel ID 6 distal wrist actuator (Robotis, Seoul, Republic of Korea) as the controlled isolated test bed required to expose deployment-layer effects with reduced multi-body coupling, showing that the final tuned enhanced implementation reduces the mean absolute error from $10.476^{\circ }$ to $1.054^{\circ }$ and the internal command saturation ratio from 0.450 to 0.012 relative to the direct baseline implementation under the same reported hardware setting.

## 2. Related Work

Robot-manipulator control remains difficult because the controller must simultaneously cope with nonlinear dynamics, unknown inertia and gravity terms, actuator uncertainty, friction, saturation, and sensing imperfections. Classical PID control remains attractive in this setting because it is compact, interpretable, and computationally inexpensive, but conventional PID tuning does not by itself solve unknown dynamics or unknown control-direction effects. To overcome these limitations, neural- and RBF-based adaptive schemes have been widely used to approximate uncertain robot dynamics and to make PID-type controllers self-adjusting, including constrained neural-adaptive PID control [8], learning-based actor–critic PID tuning [9], and nonlinear RBF–PID designs validated on real platforms [10]. A parallel line of work has concentrated on the actuation- and constraint-related effects that dominate physical deployment: input saturation and bounded actuation [11,12], joint-space and full-state constraints [13,14], dead-zone and disturbance effects handled through disturbance observers [15], and unknown control direction [5]. Recent work on Bayesian controller tuning, intelligent motor-controller self-tuning, and real-platform validation further reinforces the need to evaluate controllers with the physical actuator, sensing, and command limits present in the loop rather than relying only on simulation-level behaviour [10,16,17,18,19]. Together these studies show that adaptive PID-type control is mature in theory, yet most results are established analytically or in simulation rather than on directly low-cost hardware.

This is the motivation behind the Nussbaum-function PID controller proposed by Rahimi Nohooji and Voos [3], the direct theoretical starting point of the present study, which unifies the simplicity of a PID-like structure with the adaptive sign-handling capability of a Nussbaum function. Their control law links the proportional, integral, and derivative gains through a small set of parameters and introduces the intermediate signal Ψ(t), an RBF neural approximation term, and the scalar adaptation state ζ(t). The Nussbaum function $N\left(\zeta \right)=\zeta^{2}\cos\left(\zeta \right)$ then modulates the control action so that the method can address unknown control direction without requiring an exact model of the manipulator dynamics. This is an important advantage because incorrect control-direction assumptions can destabilise a robot, whereas the Nussbaum mechanism provides a mathematically grounded way to adapt the effective control sign [4,20].

The source paper also has a practical design attraction: it attempts to reduce tuning complexity. Instead of independently tuning a full PID gain set, the method uses linked gains and adaptive laws so that gain determination is partly automated. The authors prove boundedness of the closed-loop signals using Lyapunov analysis and then validate the theoretical framework through numerical simulation of a two-link robot manipulator in the vertical plane. In that simulation, the desired trajectories are sinusoidal, the robot parameters are known for generating the simulated plant, and the reported plots show position and velocity tracking, bounded generalised error Ψ(t), and bounded control input.

This leaves a clear deployment gap. The Rahimi Nohooji–Voos paper establishes a useful theoretical controller and demonstrates it numerically, but it does not show what happens when the same adaptive NPID structure is mapped into a real low-cost actuator through direct current commands. Real hardware introduces effects that are absent or idealised in the simulation: encoder quantisation, low-speed friction, command sign conventions, current saturation, current slew limits, communication timing, and actuator dead zones. These effects are especially important for Nussbaum control because the adaptation state ζ can drift into regions where N(ζ) becomes small or changes sign. In a purely numerical setting this behaviour is handled inside the theoretical analysis, but on a current-limited motor it can cause loss of practical control authority. This limitation is closely related to the broader control literature on input saturation and bounded actuation, where mathematical torque commands must still be converted into commands that a real actuator can deliver [11,21]. A key distinction of the present work is the layer at which these effects are addressed: whereas the studies above treat saturation, dead zones, and constraints as terms inside the controller-synthesis and stability analysis, the present paper treats them at the deployment layer of a directly current-driven low-cost actuator, where the realisable command, not the mathematical torque, sets the achievable behaviour.

The present paper is therefore positioned as a real-hardware continuation of the NPID idea rather than as a replacement for it. The goal is to preserve the interpretable NPID core while adding the hardware-facing regularisation needed for sustained physical operation. This includes bounded adaptation management, low-speed velocity-reference feedforward, tail-region damping, current-command mapping, and safety limits. Since these parameters interact strongly and each closed-loop trial is expensive and potentially unsafe, the tuning itself is treated as part of the experimental method rather than as manual trial and error. Optuna is used as the experimental tuning framework because its define-by-run search spaces and TPE sampler [7] allow the controller to be tuned against real-hardware criteria rather than only against ideal simulation metrics. This decision also follows a growing trend in controller tuning, in which Bayesian and safe optimisation methods are increasingly used when closed-loop tests are expensive, nonlinear, and potentially unsafe [16,17,18,22,23,24,25].

The research gap addressed by this paper can now be stated precisely: the original NPID controller is theoretically attractive because it is compact, model-light, PID-like, and able to address unknown control direction, but its behaviour on a directly current-driven real manipulator actuator has not been characterised. This study fills that gap by implementing the controller on a Niryo NED3 Pro actuator, characterising a deployment-layer degradation mechanism in the direct baseline implementation, and evaluating an enhanced implementation on an isolated single joint (Dynamixel ID 6); the rationale for this controlled single-joint design, which establishes the secure hardware foundation for a subsequent whole-arm study, is detailed in Section 3.

## 3. Materials and Methods

### 3.1. Experimental Platform and Evidence Pipeline

Experiments were conducted on a Niryo NED3 Pro manipulator using direct Dynamixel current-command control. The manuscript evidence is deliberately concentrated on a single actuator, namely Dynamixel ID 6 (Niryo J5) in the distal wrist chain, used here as the controlled, decoupled test bed of the study.

The Nussbaum adaptation mechanism interacts with several hardware-induced effects whose individual contributions are extremely difficult to separate when the controller is exercised on a full multi-degree-of-freedom arm. These effects include unmodelled Coulomb and viscous friction at the actuator, severe encoder quantisation at the gearbox output, current-saturation and slew limits in the Mode 0 current loop, communication latency on the Dynamixel bus, and the dead-band introduced by the low-level current driver near zero command. On a multi-joint arm these effects are superimposed on the time-varying Coriolis, centrifugal, and gravity terms of (1), so that a single tracking-error trace at any joint reflects a sum of (i) the Nussbaum adaptation transient, (ii) the hardware-oriented regularisation studied here, (iii) the actuator and sensing non-idealities, and (iv) the multi-body coupling. Isolating the distal wrist joint reduces the dominant contribution of term (iv), because the upstream joints are held at a fixed configuration during every reported run, the wrist payload is fixed, and the gravity component at the operating centre is approximately constant. This design does not eliminate all residual joint-level effects: friction, backlash, compliance, cable and gearbox effects, gravity residuals, and load-dependent behaviour may still influence the same actuator. It does, however, reduce the dominant multi-body coupling confound sufficiently for the remaining residual to be interpreted primarily in terms of the Nussbaum mechanism and the actuator/sensing chain, which is exactly the deployment-layer behaviour this paper aims to characterise.

This single-joint study is the necessary first step of a multi-stage evaluation programme: characterise and improve the deployment-layer behaviour of an adaptive controller on a single decoupled joint, establish the safety envelope, the regularisation architecture, and the hardware-aware Optuna scoring, and then carry the resulting controller forward to whole-arm trajectory tracking in subsequent work. The Dynamixel ID 6/Niryo J5 actuator is well suited to this role: it sits at the distal end of the kinematic chain, so its dynamics are dominated by its own rotor inertia and friction rather than by the rest of the arm; it is the actuator most exposed to encoder-quantisation and low-speed effects in the NED3 Pro platform; and it is the actuator for which sustained current-mode operation is most demanding because the available current ceiling is modest relative to the demanded torque at low speeds. The paper uses the dual identifier “Dynamixel ID 6/Niryo J5” to avoid ambiguity between actuator IDs and whole-arm joint numbering conventions used in earlier working notes.

**Figure 2:**
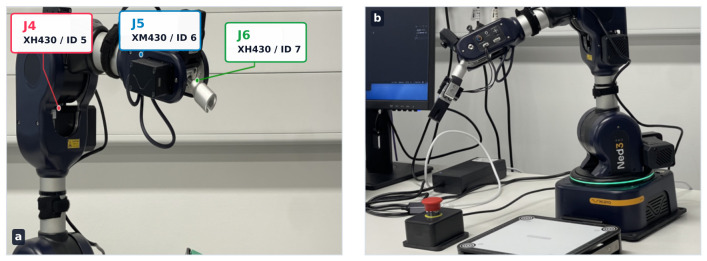
Real-hardware platform used in the study: (**a**) annotated distal-actuator map showing the J4–J6 chain and the DYNAMIXEL IDs, including the tested ID 6/J5 actuator; and (**b**) the Niryo NED3 Pro manipulator used for the validation experiments.

The controller was executed outside the higher-level ROS command path for the final evidence runs. This decision was made after early experiments showed that command-state conflicts and timing jitter in the middleware path could obscure the low-level behaviour of the adaptive law. The final evidence loop therefore logged the reference position, measured position, filtered velocity, tracking error, intermediate Nussbaum variables, commanded current, measured current, and saturation indicators in a single direct loop. Figure 2 shows the annotated distal actuator map and the robot used to ground the experiments in the physical platform.

The locked-centre sinusoidal reference family was chosen to probe the dominant deployment stresses without leaving the safe repeatable operating region of the distal wrist. The $140^{\circ }$ centre was selected during preliminary checks because it kept the tested actuator away from joint guards and allowed repeatable start-centred operation. The $10^{\circ }$, 0.05Hz trajectory is the headline long-horizon case because it stresses low-speed reversal behaviour, encoder quantisation, and current dead-band effects while remaining slow enough to separate adaptation drift from bandwidth limits. The $40^{\circ }$ cases test the same controller family over a larger excursion and larger reversal energy. The 0.5Hz cases then increase the reversal rate while remaining within the usable envelope identified for this low-cost current-command stack; higher-frequency 1.0–3.0Hz runs are reported separately as bandwidth-limit probes rather than as in-envelope validation.

Table 1 summarises the main experimental protocol used for the headline real-hardware validation.

### 3.2. Baseline Nussbaum-PID Formulation

The manipulator model adopted in the source NPID work [3] is

(1) $$M\left(q\right)\ddot{q}+C\left(q,\dot{q}\right)\dot{q}+G\left(q\right)=\tau ,\;\tau =\kappa \;u,$$

where $q\in R^{n}$ is the joint position vector, M(q) is the symmetric positive-definite inertia matrix, $C\left(q,\dot{q}\right)\dot{q}$ collects Coriolis and centrifugal terms, G(q) is gravity, τ is the applied joint torque, *u* is the control input, and κ is an unknown control-direction matrix. In the real-hardware deployment of Section 4, unmodelled friction, encoder quantisation, communication latency, and current-command nonlinearities additionally act on the joint; these are treated explicitly at the deployment layer rather than absorbed into (1).

For tracking, the joint-level error signals are

(2) $$e=q_{d}-q,\;\dot{e}={\dot{q}}_{d}-\dot{q},\;e_{I}\left(t\right)=\int_{0}^{t}e\left(\xi \right)\;d\xi ,$$

and the source formulation defines the generalised intermediate variable

(3) $$\Psi \left(t\right)=2\gamma e\left(t\right)+\gamma^{2}e_{I}\left(t\right)+\dot{e}\left(t\right),\;\gamma >0,$$

which couples proportional, integral, and derivative tracking error into a single signal. The role of Ψ is established in [3]: if Ψ(t)→0 as t→∞, then *e*, $\dot{e}$, and $e_{I}$ remain bounded and converge to zero.

For dimensional consistency in Equation (3), the parameter γ has units of $s^{-1}$. Therefore, 2γe, $\gamma^{2}e_{I}$, and $\dot{e}$ all have angular-rate units. In the implementation reported in this paper, the controller signals *q*, $q_{d}$, *e*, $e_{I}$, and $\dot{e}$ were represented in degrees, degree-seconds, and degrees per second, respectively, because these are the native units used in the logged actuator data and in the reported tracking-error metrics. The same mathematical structure can be implemented using radians if all signals and gains are rescaled consistently. However, the optimised parameters reported in this paper are degree-basis implementation parameters and should not be transferred directly to a radian-basis controller without the corresponding unit conversion.

To avoid ambiguity between the source theoretical formulation and the implemented single-joint controller, the RBF input used in this hardware implementation is defined explicitly as

(4) $$\chi ={\left[\begin{array}{ccc} \Psi & q & \dot{q} \end{array}\right]}^{⊤}.$$

The corresponding RBF approximation is $F\left(\chi ;\psi \right)=\psi^{⊤}\phi \left(\chi \right)$, with Gaussian basis functions

(5) $$\phi_{i}\left(\chi \right)=\exp\;\left(-\frac{1}{2}{\left(\chi -c_{i}\right)}^{⊤}B_{i}^{-1}\left(\chi -c_{i}\right)\right),$$

where $c_{i}$ is the centre of the *i*th basis function, and $B_{i}$ is its covariance matrix. This implementation-level regressor differs from the broader source-paper notation $x={\left[e^{⊤},{\dot{e}}^{⊤},q^{⊤},\Psi^{⊤}\right]}^{⊤}$. The reduced regressor χ was used because the present validation is a single-joint hardware deployment study and because Ψ already combines the proportional, integral, and derivative tracking-error information used by the controller. All RBF centres, widths, and tuned gains reported in this paper therefore correspond to the implemented regressor χ, not to the full source-paper vector *x*. The adaptive gain correction is

(6) $$\kappa_{\Delta }\left(t\right)=-\alpha \;\hat{\psi }{\left(t\right)}^{⊤}\phi \left(\chi \right),$$

and the RBF weight update is

(7) $$\dot{\hat{\psi }}\left(t\right)=-\Gamma \;\left(\alpha {∥\Psi (t)∥}^{2}\phi \left(\chi \right)+\sigma_{m}\;\hat{\psi }\left(t\right)\right),$$

with α>0, $\Gamma =\Gamma^{⊤}>0$, and $\sigma_{m}>0$ the σ-modification leakage term (denoted σ in [3]; renamed here only to avoid collision with the RBF width symbol). Throughout the rest of the manuscript, $\sigma_{m}$ refers only to this leakage coefficient; the RBF spread is represented by $B_{i}$ in Equation (5) or by the reported RBF width parameter, and these symbols are not interchangeable.

The Nussbaum function used throughout this work is

(8) $$N\left(\zeta \right)=\zeta^{2}\cos\left(\zeta \right).$$

and the associated Nussbaum gain is $K_{N}\left(\zeta \right)=-N\left(\zeta \right)$. Although this function satisfies N(0)=0, this is a property of the Nussbaum function rather than an initial condition for the adaptation state. The hardware implementation therefore used the non-zero initial adaptation state

(9) $$\zeta \left(0\right)=\zeta_{0}=0.8856929670,$$

which was selected during the offline Optuna tuning process together with the remaining deployment parameters, rather than chosen manually after the validation run. The general source control law is

(10) $$u\left(t\right)=\left(k_{\pi }+\kappa_{\pi }\right)K_{N}\left(\zeta \right)\;e+\left(k_{\iota }+\kappa_{\iota }\right)K_{N}\left(\zeta \right)\;e_{I}+\left(k_{\Delta }+\kappa_{\Delta }\right)K_{N}\left(\zeta \right)\;\dot{e},$$

and the source then imposes the linking relations

(11) $$k_{\Delta }=\frac{k_{\pi }}{2\gamma }=\frac{k_{\iota }}{\gamma^{2}},\;\kappa_{\Delta }\left(t\right)=\frac{\kappa_{\pi }\left(t\right)}{2\gamma }=\frac{\kappa_{\iota }\left(t\right)}{\gamma^{2}},$$

which yield the characteristic polynomial $s^{2}+2\gamma s+\gamma^{2}={\left(s+\gamma \right)}^{2}$. For γ>0, this polynomial is Hurwitz, and the control law reduces to the compact form   

(12) $$u_{\mathrm{pure}}=-\left(k_{\Delta }+\kappa_{\Delta }\right)N\left(\zeta \right)\;\Psi ,$$

with adaptation-state dynamics

(13) $$\dot{\zeta }=\Psi^{⊤}\left(k_{\Delta }+\kappa_{\Delta }\right)\Psi =\left(k_{\Delta }+\kappa_{\Delta }\right){∥\Psi ∥}^{2}.$$

Equation (13) is mathematically concise and was implemented directly in the initial hardware baseline. In the reported real-motor tests, this direct implementation showed a deployment-layer degradation mechanism. Since ${∥\Psi ∥}^{2}\geq 0$, ζ can accumulate over long runs when $k_{\Delta }+\kappa_{\Delta }$ remains predominantly positive in the tested regime. As ζ approaches regions where cos(ζ) becomes small, the effective multiplicative action of N(ζ) decreases; after crossing such regions, the effective sign of the Nussbaum modulation can change. On a current-limited actuator with friction and quantised sensing, this behaviour can reduce practical control authority. This observation concerns the direct hardware transfer of the baseline law and should not be read as a contradiction of the source Lyapunov analysis.

### 3.3. Enhanced NPID Formulation

The goal of the enhanced NPID formulation was to preserve the Nussbaum core while preventing the actuator from being driven into the most damaging parts of the baseline adaptation trajectory. Three additions were kept in the final manuscript configuration. First, the ζ-law was regularised using a soft leakage term:

(14) $$\dot{\zeta }=\left(k_{\Delta }+\kappa_{\Delta }\right){∥\Psi ∥}^{2}-\lambda_{\zeta }\max\left(0,\zeta -\zeta_{\mathrm{soft}}\right).$$

This term is used here as an implementation-level adaptation governor. It does not remove the Nussbaum mechanism because N(ζ) remains in the control law. However, it is not claimed as a general replacement for the original Nussbaum adaptation law. Its purpose is to slow excessive ζ-growth in the tested actuator setting once ζ enters a range empirically associated with poor long-duration hardware behaviour.

Second, a low-speed velocity-reference feedforward term was added:

(15) $$u_{\mathrm{vref}}=k_{f}\tanh\;\left(\frac{{\dot{q}}_{d}}{v_{f}}\right),$$

where $k_{f}$ is the feedforward magnitude, and $v_{f}$ is the velocity scale used to smooth the sign transition. The term is motivated by Coulomb-friction-like low-speed hesitation, but it uses the desired velocity ${\dot{q}}_{d}$, not the measured velocity $\dot{q}$. It is therefore described here as velocity-reference feedforward rather than as a direct friction observer or measured-velocity friction compensator. This design was used to provide a smooth reversal bias without injecting measured-velocity noise into the current command.

Third, a tail-region damping term was activated only when the absolute tracking error became large enough to justify extra damping. In the implementation, the activation weight is a smoothstep map between an error threshold and a full-activation error level. In compact notation,

(16) $$a_{\mathrm{tail}}\left(|e|\right)\in \left[0,1\right],$$

where $a_{\mathrm{tail}}$ increases monotonically from 0 to 1 as |e| grows between a lower error threshold and a full-activation level through a smoothstep (cubic Hermite) interpolation, so that the additional damping stays inactive during normal small-error tracking and engages only on large excursions; the two activation thresholds are reported with the other deployment parameters in the released configuration files. The additional tail damping is

(17) $$u_{\mathrm{tail}}=-k_{td}\dot{e}.$$

The implemented enhanced NPID control signal before current mapping is therefore

(18) $$u_{\mathrm{pp}}={\mathrm{sat}}_{u_{\max}}\left(u_{\mathrm{pure}}+a_{\mathrm{tail}}\left(|e|\right)\;u_{\mathrm{tail}}+u_{\mathrm{vref}}\right).$$

Here, ${\mathrm{sat}}_{u_{\max}}\left(\cdot \right)$ denotes symmetric saturation at the internal controller limit $u_{\max}$. The variable $u_{\mathrm{pp}}$ is a dimensionless controller-side command and should not be interpreted as a joint torque. These three additions define the enhanced NPID controller studied here. Other engineering safeguards, including anti-windup, velocity filtering, saturation-aware freezing of adaptation, current slew limiting, and a joint-angle guard, remained active as the fixed deployment envelope during all real-hardware trials. They are important for reproducible execution, but they were not treated as the main scientific novelty of the manuscript.

**Figure 3:**
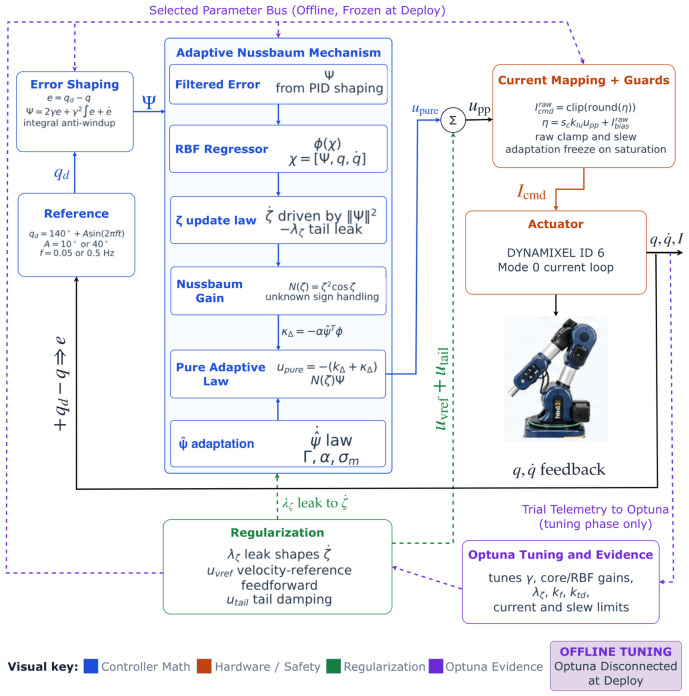
Closed-loop block diagram of the hardware-aware NPID architecture. The main chain runs from the locked-centre sinusoidal reference $q_{d}$ through error shaping (Ψ), the adaptive Nussbaum mechanism (six sub-blocks: filtered error Ψ, RBF regressor ϕ(χ), $\hat{\psi }$ adaptation, ζ update law, Nussbaum gain N(ζ), and pure adaptive law $u_{\mathrm{pure}}$), the summing junction ($u_{\mathrm{pp}}$), the current mapping and safety guards ($I_{\mathrm{cmd}}$), and the Dynamixel actuator in Mode 0 current loop, with the measured state $\left(q,\dot{q}\right)$ fed back.

Figure 3 summarises the full closed-loop architecture and the role of every equation introduced above. The signal flow runs from left to right along the main horizontal chain. The Reference block emits the locked-centre sinusoidal reference $q_{d}=140^{\circ }+A\sin\left(2\pi ft\right)$ with the validated amplitudes $A\in \{10^{\circ },40^{\circ }\}$ and frequencies f∈{0.05,0.5}Hz. The Error shaping block applies Equations (2) and (3) to produce the generalised error Ψ, with integral anti-windup acting on the integrator inside Ψ. The Adaptive Nussbaum mechanism expands its internal structure into six sub-blocks arranged in a 3×2 grid: the left column holds the filtered error (Ψ routed in from PID shaping), the $\hat{\psi }$ adaptation law of Equation (7) with hyperparameters $\Gamma ,\alpha ,\sigma_{m}$, and the pure adaptive law $u_{\mathrm{pure}}=-\left(k_{\Delta }+\kappa_{\Delta }\right)N\left(\zeta \right)\Psi$ of Equation (12); the right column holds the RBF regressor ϕ(χ) with input $\chi =[\Psi ,q,\dot{q}]$, the ζ update law ($\dot{\zeta }$ driven by ${∥\Psi ∥}^{2}$ with the $-\lambda_{\zeta }$ tail-leak input from the regularisation block), and the Nussbaum gain $N\left(\zeta \right)=\zeta^{2}\cos\zeta$ that supplies unknown-sign handling. The block-level output $\kappa_{\Delta }=-\alpha {\hat{\psi }}^{\;⊤}\;\phi$ of Equation (6) is shown along the bottom of the mechanism panel, where the RBF regressor output ϕ is combined with the adapted weights $\hat{\psi }$ to drive the pure adaptive law.

The three hardware-oriented additions are grouped in the Regularisation block at the bottom of the figure and are routed via dashed green arrows back into the controller: the soft ζ-leak $-\lambda_{\zeta }{\left[\zeta -\zeta_{\mathrm{soft}}\right]}_{+}$ is injected directly into the ζ-update sub-block of the adaptive mechanism, while the velocity-reference feedforward $u_{\mathrm{vref}}$ of Equation (15) and the tail damping $u_{\mathrm{tail}}$ of Equation (17) are summed with $u_{\mathrm{pure}}$ at the explicit Σ junction outside the mechanism panel. The summed pre-deployment command $u_{\mathrm{pp}}$ then enters the Current mapping + guards block, which applies the current mapping of Equation (19), the current and slew clamps, and the saturation-aware adaptation freeze before issuing the raw current command $I_{\mathrm{cmd}}$ to the Actuator (Dynamixel ID 6 in Mode 0 current loop). The measured joint state $\left(q,\dot{q}\right)$ is fed back to the input summing junction to close the position loop.

A second, offline loop is shown in dashed purple. The Optuna tuning and evidence block consumes per-trial telemetry from the actuator during the tuning phase only, and distributes the selected hyperparameters along the selected parameter bus to every tunable block: γ in error shaping, the core/RBF gains $\left(k_{\Delta },\alpha ,\Gamma ,\sigma_{m},\mathrm{RBF}\right)$, the regularisation parameters $\left(\lambda_{\zeta },k_{f},k_{td}\right)$, and the current and slew limits in the current-mapping block. The amber OFFLINE TUNING badge at the top-right of the figure, the (offline, frozen at deploy) qualifier on the parameter bus, and the (tuning phase only) qualifier on the telemetry arrow all reinforce that this Optuna loop is not part of the deployed real-time controller: once the parameter set has been selected, the Optuna bus and tuning telemetry are disconnected, the parameters are frozen, and the controller runs as a fixed-parameter real-time loop. The colour key at the bottom of the figure separates the four functional concerns: controller math (blue), hardware/safety (orange), regularisation (green), and Optuna evidence (purple).

### 3.4. Current-Command Deployment Layer

The actuator does not accept a torque command directly. The dimensionless controller command $u_{\mathrm{pp}}$ is therefore mapped into raw Dynamixel current-command counts as

(19) $$I_{\mathrm{cmd}}^{\mathrm{raw}}=\max\;\left(-I_{\max}^{\mathrm{raw}},\min\;\left[I_{\max}^{\mathrm{raw}},\mathrm{round}\left(s_{c}k_{Iu}u_{\mathrm{pp}}+I_{\mathrm{bias}}^{\mathrm{raw}}\right)\right]\right).$$

where $k_{Iu}$ maps the dimensionless controller command into raw current counts, $I_{\mathrm{bias}}^{\mathrm{raw}}$ is a raw-count bias, $I_{\max}^{\mathrm{raw}}$ is the raw current clamp, and $s_{c}\in \left\{-1,+1\right\}$ is the experimentally identified command-sign convention. For the final manuscript configuration, $s_{c}=-1$, $k_{Iu}=210$, $I_{\mathrm{bias}}^{\mathrm{raw}}=-0.1296542137$, and $I_{\max}^{\mathrm{raw}}=39$. Since the raw Dynamixel current command is integer-valued, the small optimised bias $I_{\mathrm{bias}}^{\mathrm{raw}}=-0.1296542137$ has no independent physical meaning except through its effect on rounding close to integer thresholds. It is retained here only for exact reproducibility of the released configuration selected by Optuna. In practice, this value is approximately equivalent to a zero raw current count.

If a physical current value is required, the raw command can be converted using the current least-significant bit of the tested Dynamixel actuator,

(20) $$I_{\mathrm{cmd}}^{\mathrm{mA}}=c_{I}I_{\mathrm{cmd}}^{\mathrm{raw}},$$

where $c_{I}$ is the model-specific current scale in mA/count. The deployment configuration used the XM430-W350 current-register convention for the tested Dynamixel actuator, for which the project configuration recorded $c_{I}\approx 2.69\;\mathrm{mA}/\mathrm{count}$. Therefore, the clamp $I_{\max}^{\mathrm{raw}}=39$ corresponds to approximately $I_{\max}^{\mathrm{mA}}\approx 39\times 2.69=104.9\;\mathrm{mA}$. The quantitative controller claims in this paper are nevertheless reported in raw Dynamixel current units and internal command units because those are the quantities logged by the deployment layer. The sign must be validated experimentally; it cannot be copied blindly from a different controller family.

The command sent to the motor was also subject to current clamping and a first-order slew constraint. Although these safety layers are conceptually simple, they are crucial for real-hardware credibility because they define the part of the theoretical control law that the actuator can actually realise.

### 3.5. Theoretical Scope of the Implemented Controller

The baseline NPID controller follows the theoretical formulation in the source work, where boundedness is established under the assumptions of the considered manipulator model and the corresponding adaptive control law. The enhanced controller implemented in this paper includes hardware-facing components, namely, soft adaptation-state leakage, low-speed velocity-reference feedforward, tail-region damping, current clamping, slew-rate limiting, anti-windup, and saturation-aware adaptation handling. These components are introduced for real-motor deployment and are not claimed here as a new Lyapunov-based theoretical extension of the source controller. Therefore, the claims of the present paper are restricted to experimentally observed bounded operation under the reported actuator, reference trajectory, operating region, and safety envelope. The objective is to characterise and improve practical deployability, not to replace the original theoretical analysis.

A clarification on the role of the Nussbaum mechanism in this single-joint study is warranted, because it pre-empts a natural objection. On the isolated Dynamixel ID 6 test bed, the effective command sign is in fact identifiable, and indeed the deployment layer fixes it to a constant value ($s_{c}=-1$ in Equation (19)). One might therefore ask why a Nussbaum-gain controller, whose defining purpose is to resolve an *unknown* control direction, is exercised at all on a joint whose direction is known. The answer is methodological. The unknown-sign capability of the Nussbaum law is not the property under test in this paper; it is the property the source controller will be relied upon to provide in the multi-joint and variable-transmission settings targeted in subsequent work, where effective control-direction and low-level command-mapping uncertainty genuinely arise once joints are coupled, reconfigured, or driven through gear trains of mixed convention. Before that sign-handling capability can be trusted on real hardware, the *deployment dynamics* of the very same adaptation law—the growth of ζ, the behaviour of $N\left(\zeta \right)=\zeta^{2}\cos\left(\zeta \right)$ near its critical region, and the interaction of both with current limits and friction—must first be characterised in the cleanest practical setting. A single decoupled joint with a known, fixed sign is precisely that setting: it reduces the influence of multi-body coupling and removes sign ambiguity, so that the ζ-drift degradation reported in Section 4 can be interpreted as a deployment-layer effect rather than as a sign-resolution transient. In other words, the known-sign single joint is used here not because the Nussbaum gain is needed to find the sign, but because it is the controlled experiment in which the deployability of the Nussbaum adaptation mechanism can be established before its sign-handling property is exercised on harder, genuinely sign-uncertain configurations.

### 3.6. Optuna-Guided Real-Hardware Tuning

Optuna was used in this paper *only as an offline tuning framework*. It ran on a host computer connected to the robot during the parameter-search phase, drove a sequence of finite-duration real-hardware trials, and stored trial telemetry, scores, and the selected parameter set. Once the final parameter set was chosen, the Optuna loop was disconnected, and the controller ran as a fixed-parameter real-time loop. No live optimisation, online hyperparameter adaptation, or online Optuna optimisation was used during deployment; the purple Optuna paths in Figure 3 were exercised only during the tuning phase. The host computer therefore remained part of the experimental command/logging setup but not as an online optimiser during the deployed validation runs. The manual tuning was useful for initial safety checks, but it was insufficient for the final controller because several parameters interact nonlinearly: $k_{\Delta }$, γ, α, Γ, RBF width, current ceiling, slew limit, ζ-leakage, velocity-reference feedforward, and tail-region damping. Optuna was therefore used as the main search framework [7]. The implementation used Optuna’s Tree-structured Parzen Estimator (TPE) sampler with multivariate sampling enabled and a fixed random seed for reproducibility, together with a short startup phase of random trials before the TPE model was engaged. The complete tuning archive contained 79 physical closed-loop trials, of which 61 satisfied the score-validity criterion used in the archived logs. The final manuscript configuration is therefore not presented as a blind single-trial winner; instead, it is a long-run validated configuration selected from the parameter basin exposed by the cumulative search effort and then confirmed in 300 s operation. For full transparency, the 79-trial figure refers specifically to the headline $10^{\circ }$/0.05Hz archive; the additional operating points reported later for the multi-envelope and step-response evaluations required further finite-duration Optuna passes, so the total real-hardware search effort behind the paper amounts to several hundred trials, as summarised in Table 2. All passes shared the same admissibility filters and the same scoring function of Equation (21); the quantitative selection claims in this subsection are nonetheless reported only from the single 79-trial headline archive so that every headline number is traceable to one campaign rather than aggregated across heterogeneous search budgets.

The Optuna score was deliberately hardware-aware. Trials were rejected or penalised if they violated core admissibility conditions such as insufficient motion span, excessive maximum error, excessive 95th-percentile error, or excessive adaptation-state growth. For the headline archive, the operational hard filters used by the archived tuning script were: a minimum of 40 parsed samples for a usable run, a motion-span ratio $0.12\leq r_{\mathrm{span}}\leq 1.40$, maximum absolute error $e_{\max}\leq 25^{\circ }$, and 95th-percentile absolute error $P95\leq 12^{\circ }$. The saturation metric was measured as the fraction of samples with $|I_{\mathrm{cmd}}^{\mathrm{raw}}|\geq 0.98I_{\max}^{\mathrm{raw}}$; it was included in the objective rather than used as a hard rejection criterion. The ζ-state was controlled by soft penalties, with ${\left|\zeta \right|}_{\max}>20$ penalised in the headline scoring configuration and optional hard ζ-growth or $\dot{\zeta }$-P95 limits available in the same tuning code for stricter search passes. The remaining trials were scored using a weighted sum of tracking accuracy, internal command saturation ratio, effort ratio, low control activity, and soft penalties on adaptation growth. To avoid ambiguity about mixed units, the objective is written below in its implemented scaled form: error quantities are in degrees, ratios are dimensionless, and the numerical weights therefore carry the effective reciprocal units needed to make *J* a scalar score. Equivalently, the degree-valued terms can be interpreted as normalised by $1^{\circ }$, while the ratio terms are normalised by one.

(21) $$\begin{array}{cc} J= & w_{\mathrm{mae}}{\mathrm{MAE}}_{\deg}+w_{\mathrm{rms}}{\mathrm{RMSE}}_{\deg}+w_{95}P95_{\deg}+w_{\max}e_{\max,\deg}\\ \; & +w_{\mathrm{sat}}\left(10\;r_{\mathrm{sat},\mathrm{int}}\right)+w_{\mathrm{eff}}\left(5\;r_{\mathrm{eff}}\right)+w_{\mathrm{span}}\left(10\;|1-r_{\mathrm{span}}|\right)\\ \; & +w_{u}\left(10\;\Pi_{u}\right)+w_{\zeta }\Pi_{\zeta }+w_{c}u_{\max}. \end{array}$$

where $r_{\mathrm{sat},\mathrm{int}}$ is the internal command saturation ratio, $r_{\mathrm{eff}}$ is the mean absolute raw-current demand divided by $I_{\max}^{\mathrm{raw}}$, $r_{\mathrm{span}}$ is the achieved motion-span ratio, $\Pi_{u}=\max\left(0,r_{u,\min}-r_{u}\right)$ penalises insufficient controller activity, $\Pi_{\zeta }$ collects soft exceedances of the configured ζ-magnitude/growth limits, and $u_{\max}$ is the internal command clamp. For the headline archive, the active weights were $w_{\mathrm{mae}}=1.0$, $w_{\mathrm{rms}}=0.6$, $w_{95}=0.2$, $w_{\max}=0.03$, $w_{\mathrm{sat}}=1.5$, $w_{\mathrm{eff}}=0.1$, $w_{\mathrm{span}}=0.8$, $w_{u}=0.8$, $w_{\zeta }=0.1$ for the active ζ-soft-limit term, and $w_{c}=0.05$. Tail-window and step-specific weights were available in the tuning code but set to zero for the headline sinusoidal archive. This structure is closer to real experimental design than to ordinary curve-fitting because it explicitly encodes what counts as a successful and defensible hardware run.

### 3.7. Evaluation Metrics

All final evidence was assessed using fixed metrics computed from the saved CSV logs. The core metrics were mean absolute error, root-mean-square error, 95th-percentile absolute error, maximum absolute error, internal command saturation ratio, and total ζ-growth over the run. For a run with *N* logged in-loop samples and per-sample tracking error $e_{k}$, these are $\mathrm{MAE}=\frac{1}{N}\sum_{k=1}^{N}\left|e_{k}\right|$, $\mathrm{RMSE}=\sqrt{\frac{1}{N}\sum_{k=1}^{N}e_{k}^{2}}$, P95 the 95th percentile of $\left\{|e_{k}|\right\}$, $e_{\max}=\max_{k}\left|e_{k}\right|$, and the total adaptation growth Δζ=ζ(T)−ζ(0) over a run of duration *T*. The internal command saturation ratio reported throughout the paper is defined as the fraction of in-loop samples for which $|u_{\mathrm{pp}}|$ reaches the controller’s internal command clamp max_control; it therefore measures how often the adaptive law was running against its configured authority limit rather than how often the raw motor current itself touched the Dynamixel current rail. These metrics were selected because they capture complementary aspects of performance: average tracking quality, worst-case excursions, actuator stress, and long-horizon adaptation behaviour.

## 4. Results

### 4.1. Source-Paper Numerical Simulation Evidence

Before presenting the real-hardware results, it is useful to state what was already demonstrated in the source NPID paper. Rahimi Nohooji and Voos validated their controller numerically on a two-link robot manipulator moving in the vertical plane [3]. The simulation used sinusoidal desired trajectories, linked PID/Nussbaum parameters, an RBF neural approximator, and the adaptation laws for $\hat{\psi }\left(t\right)$ and ζ(t). The reported results showed position tracking, velocity tracking, bounded generalised error Ψ(t), and bounded control input under the assumptions of the source theoretical formulation.

The present paper uses that source formulation as the baseline controller for real-hardware assessment. The source-paper simulation did not include the direct current-command interface, encoder quantisation, actuator friction, command-sign identification, current saturation, or slew-rate limits of the Niryo NED3 Pro actuator. The following subsections therefore move from the original numerical setting to real physical validation. No figure from the source manuscript is reproduced here; any future reuse of source-paper figures should follow the required citation and permission process.

### 4.2. Best 300 s Enhanced Nussbaum Tracking Performance on Real Hardware

**Figure 4:**
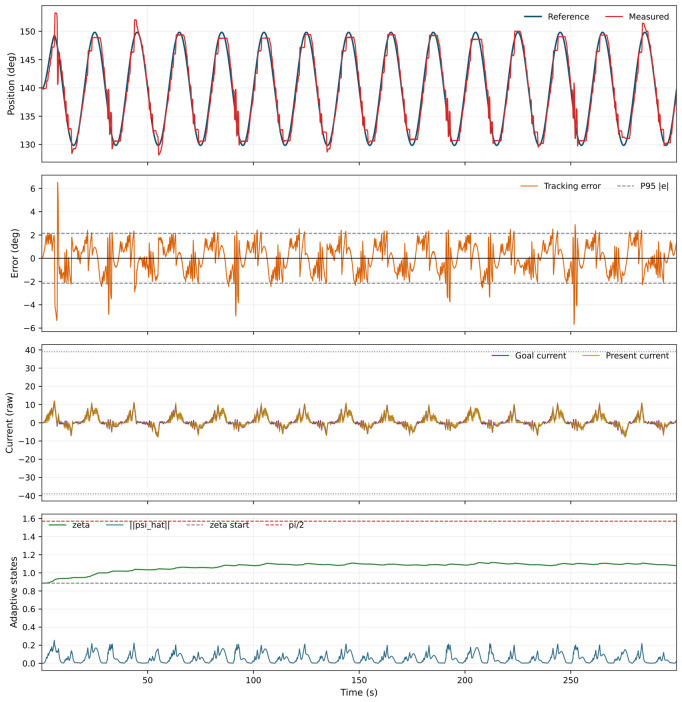
Headline configuration: $10^{\circ }$ amplitude at 0.05Hz on Dynamixel ID 6, 300s validation. Four-panel layout: position, tracking error with ±P95 lines, commanded and measured current with safety rails, and adaptive states $\zeta ,∥\hat{\psi }∥$ against $\zeta_{0}$ and π/2.

The best validated 300 s enhanced Nussbaum run in the archive is the final manuscript configuration, archived internally as break-c12a-300. Figure 4 in the multi-envelope subsection that follows renders this run in the four-panel template used throughout the Results section. The corresponding scalar performance is summarised in Table 3. The best run achieved a mean absolute error of $1.054^{\circ }$, root-mean-square error of $1.283^{\circ }$, 95th-percentile absolute error of $2.145^{\circ }$, maximum absolute error of $6.530^{\circ }$, internal command saturation ratio of 0.012, and total ζ-growth of 0.194. These figures correspond to the single best validated run (approximately 7200 in-loop samples at the 24 Hz control rate over 300 s) rather than to an ensemble average across repeated runs; this single-run mode of reporting is stated explicitly here and is revisited as a limitation in Section 5. These values support the limited practical claim of the paper: after hardware-oriented regularisation and structured tuning, the implemented NPID controller tracked a long-horizon sinusoid on the tested real actuator without returning to the degradation pattern observed in the direct baseline implementation.

Relative to the strongest pre-final velocity-reference-feedforward baseline bestnow-fric-300, the final manuscript run improved MAE by approximately 8.8%, improved the P95 error by approximately 6.0%, reduced the maximum absolute error by approximately 28.1%, and lowered the internal command saturation ratio by approximately 14.3%.

### 4.3. Multi-Amplitude and Multi-Frequency Sinusoidal Validation

To probe the operating envelope of the enhanced NPID implementation, the same controller family was retuned and revalidated on the same Dynamixel ID 6 actuator at a second amplitude ($40^{\circ }$) and at a higher frequency band (0.5Hz), in addition to the headline $10^{\circ }$/0.05Hz configuration. Table 4 reports the resulting metrics from the best validated real-hardware run at each operating point. The amplitude–frequency sweep is reported on the same joint and within the same safety envelope, so the runs are directly comparable.

The strongest two results are the $10^{\circ }$/0.05Hz headline configuration and the $40^{\circ }$/0.05Hz long-horizon run, which both retain bounded adaptation over the full 300s validation horizon. At $40^{\circ }$/0.05Hz, the larger commanded sweep increases reversal energy and slightly raises the internal command saturation ratio, but the controller still tracks the reference with a correlation of 0.992 and a small phase lag of ≈0.14 s. At $10^{\circ }$/0.5Hz the controller remains bounded and usable with near-unity amplitude ratio, although the higher reversal rate raises the internal command saturation ratio and increases mean error. The $40^{\circ }$/0.5Hz run is reported as the best short-window tracking result obtained at the high-amplitude high-rate corner; in this case the controller did not converge to a trustworthy long-horizon configuration within the search budget, and the row is therefore included as a partial-validation result rather than as a long-duration claim.

**Figure 5:**
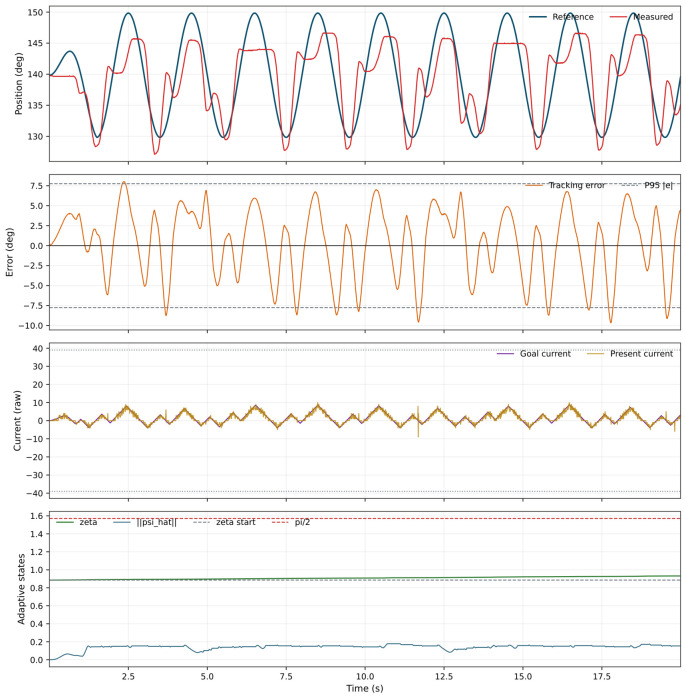
Amplitude of $10^{\circ }$ at 0.5Hz. Bounded and practically usable tracking with near-unity amplitude ratio; the higher reversal rate raises the internal command saturation ratio relative to the headline run, visible in the current panel.

**Figure 6:**
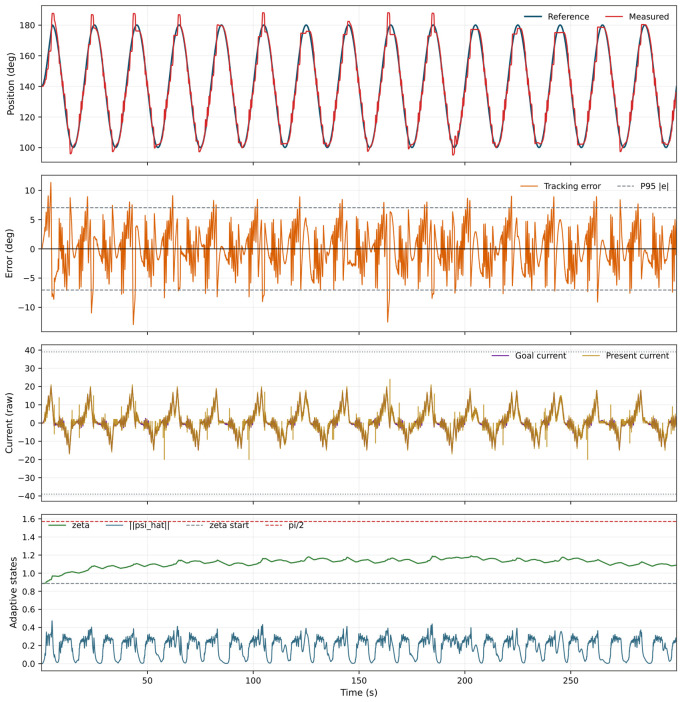
Amplitude of $40^{\circ }$ at 0.05Hz: best validated long-horizon 300s run at the larger amplitude. Tracking stays in phase with the reference and ζ remains well below π/2 for the full validation window.

**Figure 7:**
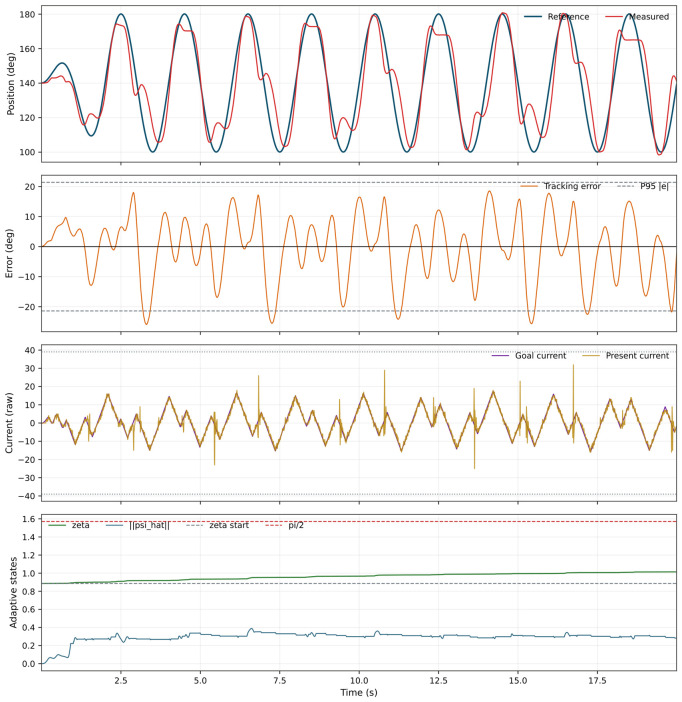
Amplitude of $40^{\circ }$ at 0.5Hz: best short-window tracking at the high-amplitude/high-rate corner. The current panel shows the larger commanded effort and the error panel shows the broader residual envelope; no trustworthy long-horizon configuration was reached within the search budget.

Figure 4, Figure 5, Figure 6 and Figure 7 render the four validated sinusoidal operating points using a single four-panel template: position (reference vs. measured), tracking error with ±P95 guides, commanded versus measured current with the safety rails, and the adaptive states ζ and $∥\hat{\psi }∥$ against the $\zeta_{0}$ and π/2 reference lines. The visual language is held constant across operating points so that the comparison can be read at a glance.

### 4.4. Bandwidth Limit of the Real Closed Loop

Two additional operating points were probed deliberately above the comfortable tracking range of the actuated joint, namely, $10^{\circ }$/1.5Hz and $10^{\circ }$/3Hz, together with high-amplitude pushes at $40^{\circ }$/1.0Hz and $40^{\circ }$/1.5Hz. These runs were not used to claim controller performance; they were used to characterise the practical bandwidth of the combined system, that is, the cascade of mechanical joint, current-command path, velocity estimation, and the enhanced Nussbaum closed loop. At $10^{\circ }$/1.5Hz, the best obtained real-hardware run remained bounded ($\mathrm{MAE}\approx 9.82^{\circ }$, $P95\approx 16.59^{\circ }$, amplitude ratio ≈1.37, phase lag ≈0.20s, correlation ≈−0.32, internal command saturation ratio ≈0.92). The negative correlation and near-clamp internal command saturation indicated that the measured response was no longer phase-aligned with the reference: the joint was responding but mostly out of phase and clipped. At $40^{\circ }$/1.0Hz, $40^{\circ }$/1.5Hz, and $10^{\circ }$/3Hz no validated tracking-quality run was obtained within the search budget; the candidates remained on the penalty floor with large persistent error and amplitude-ratio collapse.

This pattern is consistent with a classical closed-loop bandwidth limit rather than with a Nussbaum-specific failure mode. The peak reference velocity at $10^{\circ }$/3Hz reaches $2\pi \cdot 3\cdot 10^{\circ }\approx 188.5^{\circ }/s$, which in combination with the current ceiling, current slew limit, and the velocity-estimation low-pass filter places the demanded motion beyond what the present hardware/software stack can realise with phase fidelity. The implication for this paper is conservative: the multi-amplitude/multi-frequency claims in Table 4 are restricted to the ≤0.5 Hz band, and the 1.0–3Hz probes are reported as evidence of where the present low-cost stack reaches its bandwidth limit, not as failures of the controller theory.

**Figure 8:**
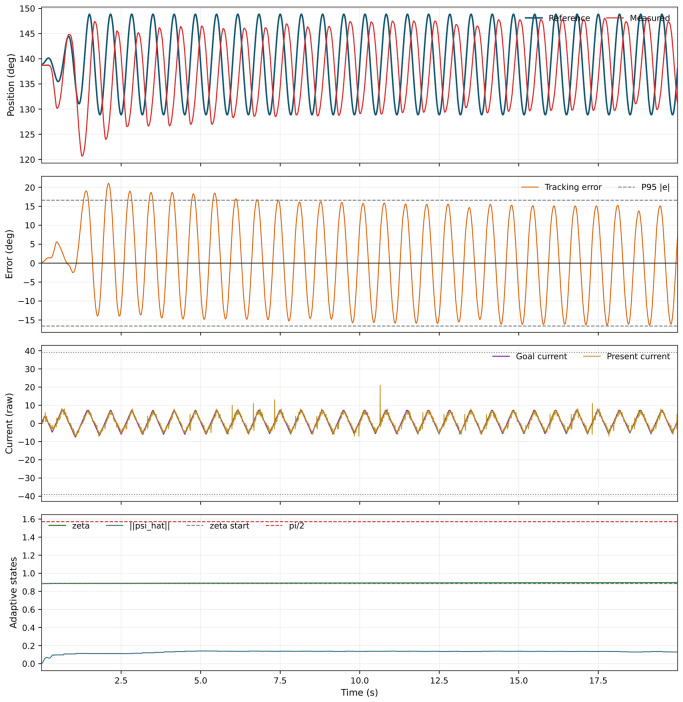
Representative bandwidth-limit probe at $10^{\circ }$/1.5Hz on Dynamixel ID 6, rendered in the same four-panel template as the in-envelope runs. The response is bounded but out-of-phase with the reference and the command sits at the internal max_control clamp for the majority of the run.

Figure 8 renders the $10^{\circ }$/1.5Hz probe as a representative bandwidth-limit run using the same four-panel template as the validated operating points, so that the failure pattern is directly comparable to the in-envelope runs: the measured trajectory is amplitude-inflated relative to the reference but largely out of phase, and the command $u_{\mathrm{pp}}$ sits at the internal max_control clamp for the majority of the run. The remaining bandwidth probes ($10^{\circ }$/3Hz, $40^{\circ }$/1.0Hz, $40^{\circ }$/1.5Hz) follow the same pattern and are documented in the Supplementary Materials. They confirm that the practical envelope boundary is set by the combined low-cost stack rather than by the controller itself.

### 4.5. Direct Baseline Versus Enhanced Nussbaum Behaviour

**Figure 9:**
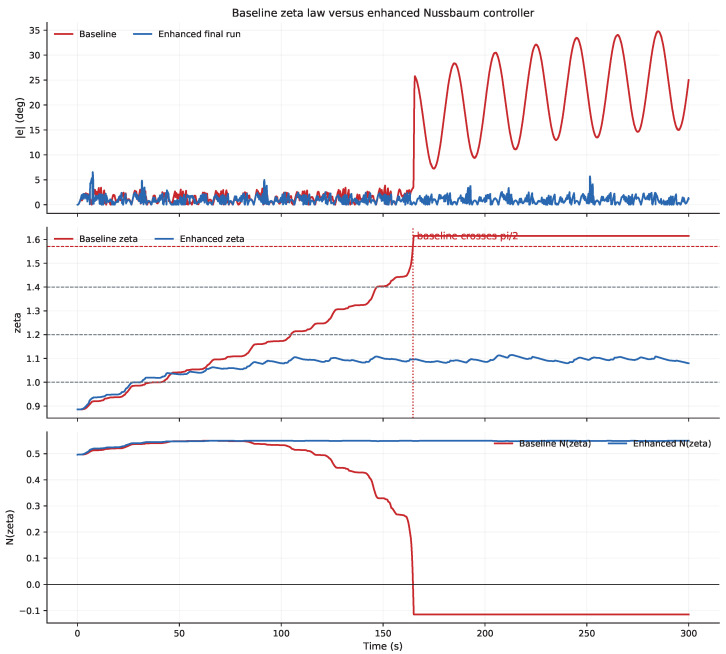
Direct baseline versus enhanced NPID comparison. The direct baseline implementation experiences rapid ζ-growth and severe loss of practical control quality, whereas the enhanced implementation retains the Nussbaum core while keeping the adaptation state away from the empirically problematic real-hardware region.

Figure 9 is the main explanatory figure because it shows why the direct hardware transfer of the baseline NPID formulation was not sufficient for the tested current-driven actuator. The baseline run exhibits rapid accumulation of ζ, high internal command saturation, and a loss of practical control authority as the Nussbaum term approaches its critical region. The enhanced formulation run, by contrast, keeps the adaptation state in a safer operating band while preserving the overall Nussbaum-driven control structure.

In the archived baseline evidence, the first critical event occurs when ζ crosses the vicinity of π/2, where cos(ζ) is close to zero. At that point, the multiplicative effect of $N\left(\zeta \right)=\zeta^{2}\cos\left(\zeta \right)$ collapses, and the effective control action weakens drastically even though the position error may still be large. Once ζ moves further, the effective sign of the Nussbaum modulation can reverse, which is especially damaging on a current-limited actuator. This is the core physical reason why the baseline configuration recorded a mean absolute error of $10.476^{\circ }$ and an internal command saturation ratio of 0.450.

### 4.6. Critical-Region Zoom During Direct Baseline Degradation

**Figure 10:**
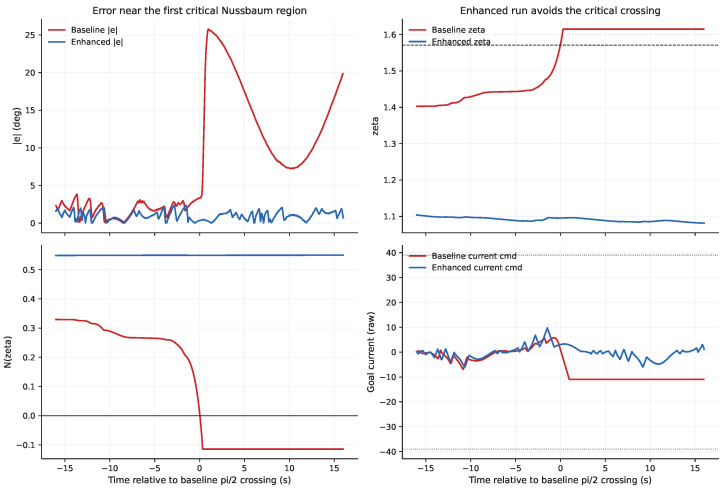
Critical-region zoom centred on the direct baseline ζ≈π/2 event. The direct baseline run undergoes simultaneous weakening of N(ζ), loss of effective current action, and growth in tracking error, whereas the enhanced run remains outside the observed critical region.

The long-run comparison in Figure 9 shows the global contrast between the baseline and enhanced controllers, but the failure mechanism becomes much clearer when the first critical event is viewed locally. Figure 10 zooms into the time window centred on the baseline ζ≈π/2 crossing. In that neighbourhood, the baseline controller shows simultaneous collapse of useful Nussbaum modulation, growth in tracking error, and loss of current-command effectiveness. The enhanced controller, by contrast, stays away from this critical crossing and therefore preserves useful control authority.

### 4.7. Ablation Across Enhanced Nussbaum Variants

**Figure 11:**
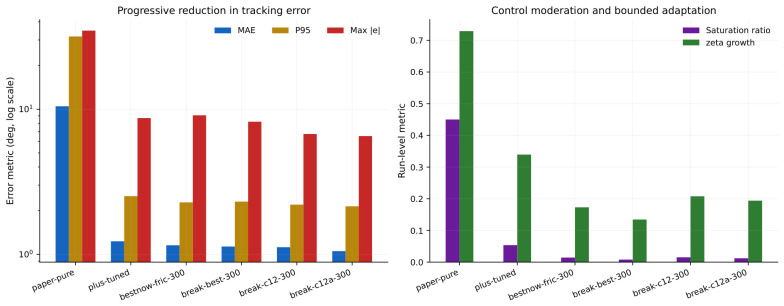
Ablation across archived Nussbaum variants. The progression shows that the enhanced-design regularisation and tuning were cumulative rather than cosmetic: error and saturation both fell substantially from the baseline NPID run to the final 300 s result.

Figure 11 summarises the progression from the initial usable enhanced Nussbaum configuration to the final manuscript run. The ablation shows that no single engineering addition was sufficient on its own. Instead, the final result emerged from a sequence of practical corrections: first making the controller move reliably, then reducing saturation, then improving low-speed behaviour via velocity-reference feedforward, and finally suppressing tail-region spikes.

This progression also reduces the risk of presenting the final controller as a one-off tuned curve. It shows a traceable path from direct baseline degradation to refined long-duration operation under the reported hardware constraints.

Two points should be stated plainly when reading Table 3, so that the contribution of each component is not overstated. First, the single largest improvement over the direct baseline is recovered once the basic deployment safeguards and low-speed velocity-reference feedforward are in place: mean absolute error falls from $10.476^{\circ }$ (direct baseline) to $1.231^{\circ }$ at the first enhanced configuration and to $1.156^{\circ }$ once velocity-reference feedforward is added, while the internal command saturation ratio drops from 0.450 to 0.014. The subsequent Nussbaum-specific regularisation and structured tuning (soft ζ-leakage, tail-region damping, and the final parameter basin) then contribute a further, smaller, but consistent refinement to $1.054^{\circ }$ mean error, with the clearest gains in the worst-case and stress metrics rather than in the average: the maximum absolute error improves from $9.081^{\circ }$ to $6.530^{\circ }$ (≈28%) and the internal command saturation ratio from 0.014 to 0.012. The honest interpretation is therefore that deployment-layer engineering and velocity-reference feedforward account for most of the recovery of average tracking, whereas the adaptation-management terms primarily contain the long-horizon tail behaviour and bounded ζ-growth that the direct baseline could not sustain. Second, the comparisons in Table 3 are made *within* the NPID family; the present study does not include a matched non-adaptive classical PID (or PID-plus-velocity-reference-feedforward-without-Nussbaum) baseline on the same actuator. The ablation should accordingly be read as isolating the contribution of each addition relative to the direct baseline implementation, not as a claim of superiority over a well-tuned classical controller. A matched classical-PID reference under the identical current-command deployment layer and safety envelope is a clear and necessary item for the multi-joint continuation of this work.

### 4.8. Optuna Search Evidence and Valid-Trial Trade-Off Landscape

**Figure 12:**
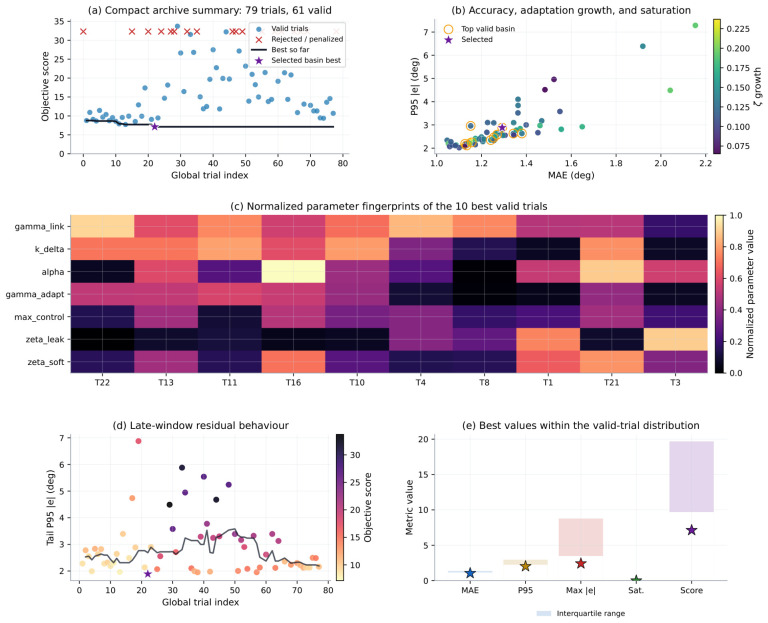
Optuna valid-trial trade-off landscape with a compact archive summary. Panel (**a**) shows the objective-score progression across the full 79-trial real-hardware archive, including the best-so-far trend. The remaining panels focus on the valid-trial subset: accuracy–adaptation trade-offs, internal command saturation, late-window residual behaviour, and normalised parameter fingerprints of the top-performing valid configurations. The merged figure preserves the readable visual style of the original valid-trial analysis while adding the minimum archive-level evidence needed to show that the final controller was selected from a structured hardware search rather than from a single favourable run.

Figure 12 combines the readable valid-trial trade-off view with a compact summary of the full Optuna archive. The archive panel shows the objective-score progression across the 79 real-hardware trials, while the remaining panels focus on the valid-trial structure: accuracy–adaptation trade-offs, internal command saturation, late-window residual behaviour, and the normalised parameter fingerprints of the best-performing valid configurations. Scientifically, this combined view shows that the selected controller was not chosen from a single favourable run but from a structured hardware search in which tracking accuracy, bounded adaptation growth, actuation limits, and long-window persistence were considered jointly. The figure supports the main methodological claim of the paper: hardware-aware tuning is part of the deployment evidence, not merely a curve-fitting step.

The practical lesson from the archive is that Optuna was not merely a convenience tool. It was the main mechanism that converted a controller with several tightly coupled nonlinear parameters into a reproducible real-hardware workflow. The search also exposed that short runs could be misleading: some candidates looked promising early but later failed in 120–300 s operation because the ζ-dynamics were still too aggressive. That is why the final manuscript run was validated at 300 s even after shorter trials had already identified attractive parameter regions.

### 4.9. Cycle-Level Residual Behaviour

**Figure 13:**
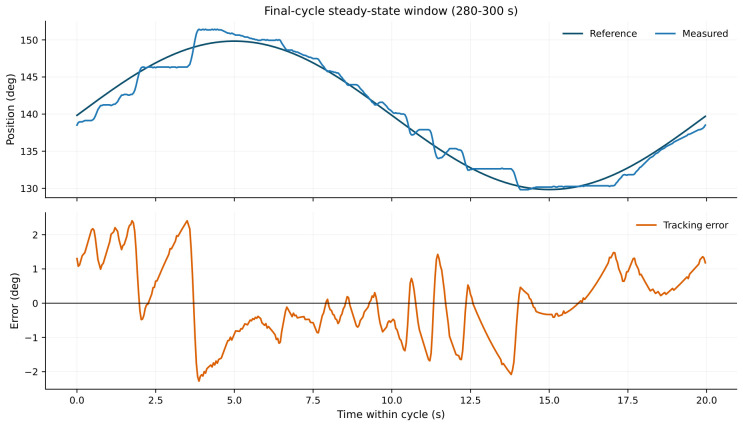
Cycle-level close-up from the final 300 s validation run. Residual error remains concentrated near reversal regions, indicating that the remaining limitation is primarily low-speed real-actuator behaviour rather than a collapse of the overall control law.

The final run achieves low aggregate error, but its residual is not perfectly smooth. Figure 13 zooms into a clean late-cycle window from the final run to show the residual behaviour that still remains after tuning. The dominant residual error occurs near reversal regions, where low-speed friction, quantised feedback, and the non-ideal realisation of very small current corrections remain coupled.

This is consistent with the design logic of the final enhanced NPID controller. The controller no longer fails through large-scale ζ-drift, but it still cannot completely remove the physical low-speed behaviour of the actuator under bounded current commands.

### 4.10. Step-Test Protocol and Real-Hardware Step Results

The same enhanced NPID controller was also evaluated using the project’s step-capable direct runner and step-aware metrics. Step sequences are scientifically complementary to the sinusoidal evidence because they separate instantaneous gain effects from the accumulated memory of the adaptation state ζ. Two step amplitudes were tested on Dynamixel ID 6, both starting from the $140^{\circ }$ operating centre: a $15^{\circ }$ step (target $125^{\circ }$) and a $40^{\circ }$ step (target $100^{\circ }$), both shaped with a short smoothstep ramp. Table 5 reports the measured step metrics. The post-1.5s and tail figures are reported separately to isolate the transient peak from the post-settling residual.

For both step amplitudes, the controller reaches and holds the demanded target with sub-degree mean error and sub-half-degree tail error, indicating that the enhanced implementation also handles abrupt reference changes without releasing the adaptation state into the empirically problematic region observed in the direct baseline. The $40^{\circ }$ step exhibits a larger transient peak error (Max $∼\;7.6^{\circ }$) than the $15^{\circ }$ step because the demanded acceleration is larger, but the post-settling behaviour remains tight in both cases.

**Figure 14:**
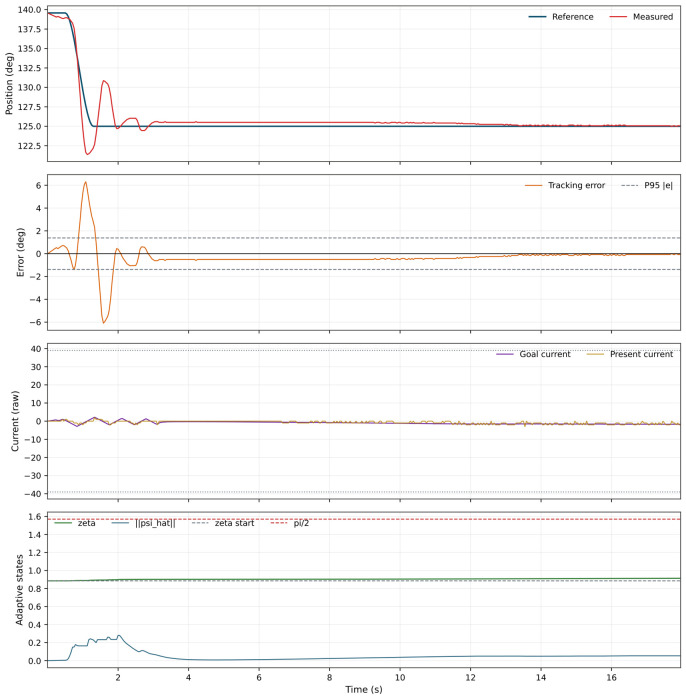
Real-hardware $15^{\circ }$ step ($140^{\circ }\to 125^{\circ }$) on Dynamixel ID 6, plotted with the same four-panel template as the sinusoidal runs. The position panel shows the smoothstep approach to the target, the error panel collapses inside ±P95 well before the settle window, and the adaptive states remain bounded throughout the transient.

**Figure 15:**
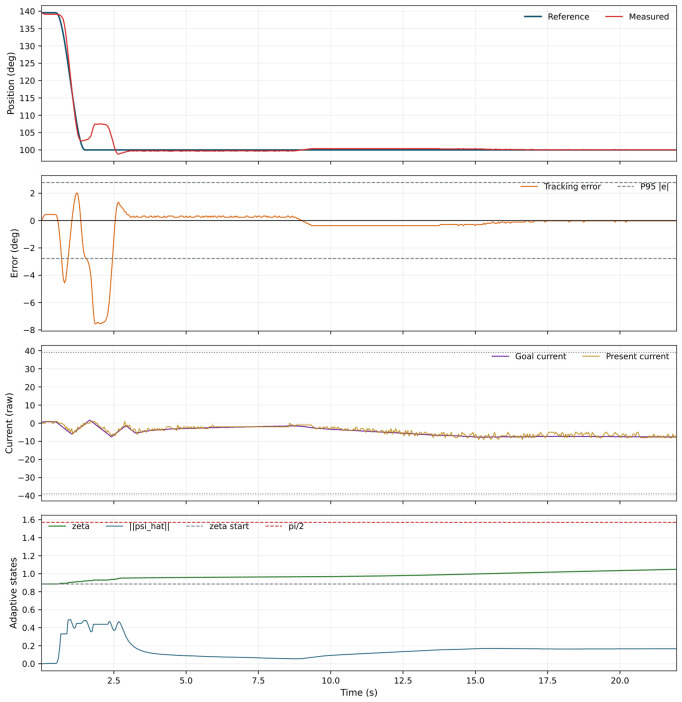
Real-hardware $40^{\circ }$ step ($140^{\circ }\to 100^{\circ }$) on Dynamixel ID 6. The larger demand produces a larger transient current and a higher peak error than the $15^{\circ }$ case, but the post-settling residual remains tight and ζ does not approach the π/2 critical line.

Figure 14 and Figure 15 visualise these two step experiments using the same four-panel format as the sinusoidal runs, making the transient error, post-settling residual, current command, and adaptive-state behaviour directly comparable to the main trajectory-tracking evidence.

### 4.11. Final Parameter Set

Table 6 lists the final parameter set used for the headline $10^{\circ }$/0.05Hz 300s run.

## 5. Discussion

The most important scientific result of the paper is not simply that the final error became small. It is that the direct baseline and enhanced implementation runs explain why the real actuator behaves differently from the theoretical idealisation. The direct baseline NPID implementation degraded in practice not because the source mathematics was contradicted, but because the adaptation state ζ accumulated under real-hardware non-idealities until the multiplicative effect of N(ζ) became physically weak in the tested actuator setting. Once current limits, low-speed friction, quantised sensing, and command-rate constraints are present, the direct transfer of the paper-level control law may not preserve the behaviour expected from the cleaner theoretical model.

This is where the enhanced NPID implementation becomes practically useful. The added ζ-leakage term does not remove the Nussbaum mechanism; it limits excessive growth of the adaptation state in the tested hardware setting. Likewise, the velocity-reference feedforward term offsets a specific low-speed actuator deficiency near reversals, while the tail damping term reduces local transient spikes that dominate the 95th-percentile error on long runs. For that reason, the enhanced controller should be read as a deployment-oriented regularisation of the direct baseline implementation, not as a new Lyapunov-based replacement for the source controller.

The role of Optuna is equally important in the final interpretation. Several of the decisive parameters in Table 6 interact strongly. For example, more aggressive control magnitude can improve short-term tracking while simultaneously worsening saturation or accelerating ζ-growth. Likewise, a stronger leak can reduce long-duration ζ-growth but may weaken the adaptive effect if chosen too aggressively. It would have been easy to overfit one metric by manual trial and error. The Optuna workflow reduced that risk by making the selection pressure explicit and reproducible. In that sense, Optuna was not just a helper script; it was part of the paper’s methodology.

The remaining limitation is also clear from the figures. Even the final controller retains residual irregularity near reversal regions. This suggests that the dominant remaining bottleneck is not the gross Nussbaum adaptation logic but the fine low-speed behaviour of the actuator under bounded current commands. More extensive compensation of friction, backlash, or velocity quantisation might reduce those residual spikes further, but such additions must be weighed against the desire to keep the controller close to the original NPID structure.

Viewed broadly, the paper argues for a particular research stance: when an adaptive controller is deployed on low-cost real hardware, the mapping layer, safety envelope, tuning method, and failure analysis should be reported as part of the scientific contribution. In this case, that reporting makes the difference between an attractive paper equation and a reproducible experimental controller.

### Scope and Limitations

The reported evidence is concentrated on a single decoupled actuator and a fixed operating region, by design rather than by convenience: as detailed in Section 3, the distal wrist joint was selected as the controlled isolated test bed required to characterise deployment-layer degradation of the Nussbaum adaptation mechanism while reducing the dominant influence of multi-body coupling. Within that test bed the validation envelope already spans two reference families (sinusoidal and step) and two amplitudes ($10^{\circ }$ and $40^{\circ }$) at frequencies up to 0.5Hz, together with bandwidth-limit probes at 1.0–3Hz. This is sufficient to substantiate the deployment-layer claims of the paper but, by construction, it does not yet establish multi-joint trajectory-tracking behaviour. The bandwidth-limit probes additionally show that, beyond roughly 0.5Hz on this low-cost stack, the dominant limitation is the combined bandwidth of mechanics, current path, and velocity estimation rather than the Nussbaum adaptation logic itself.

The principal limitations of the present study follow directly from this design and are stated explicitly. (i) *Single-run reporting.* The quantitative results in Table 3, Table 4 and Table 5 are taken from the best validated run at each operating point rather than from an ensemble of repeated trials; no run-to-run variance, confidence interval, or statistical significance test is reported. The headline numbers should therefore be read as representative best-case real-hardware behaviour under the stated envelope, not as statistical means, and a programme of repeated matched trials with mean ± standard-deviation reporting is required before stronger statistical claims can be made. (ii) *Absence of a non-adaptive baseline.* As noted in Section 4, the comparisons are internal to the NPID family; a matched classical PID and a PID-with-velocity-reference-feedforward reference under the identical deployment layer were not collected, so the paper does not establish whether the Nussbaum machinery outperforms a well-tuned conventional controller on this joint—only that the adaptive law can be deployed safely once its adaptation state is managed. (iii) *Scope of the test bed.* The evidence is confined to one decoupled distal-wrist actuator, a single $140^{\circ }$ operating centre, and a ≤0.5Hz tracking envelope; multi-joint coupling, payload variation, and disturbance rejection are out of scope by design. The study also does not include a systematic load-variation campaign, suspended-weight tests, or no-downtime thermal/endurance trials. Consequently, the reported robustness should be interpreted as robustness within the fixed-load, fixed-configuration, direct-current deployment envelope tested here, not as evidence that the same parameters are insensitive to payload, heating, or long-term friction drift. These limitations are deliberate consequences of the controlled single-joint methodology adopted here; they bound the claims of the paper rather than undermining them.

Each limitation maps directly onto a concrete next step. On top of the secure single-joint foundation established here, the natural continuation is to carry the same controller, safety envelope, and Optuna scoring forward to whole-arm trajectory tracking, to collect repeated matched trials across the remaining NED3 Pro joints for statistical reporting, and to add the matched classical-PID and PID-plus-velocity-reference-feedforward references under the identical current-command deployment layer so that the specific contribution of the Nussbaum mechanism on real hardware can be quantified directly. A further robustness campaign should vary the distal load, add suspended-weight disturbances where mechanically appropriate, and run repeated no-downtime trials so that friction drift and motor heating can be quantified rather than only discussed qualitatively.

## 6. Conclusions

This paper presented a real-hardware deployment study of a Nussbaum-function PID controller commanded through direct current control on a low-cost manipulator. A direct implementation of the baseline NPID law was first reproduced and shown to degrade in the tested hardware setting through cumulative ζ-growth, weakening of effective Nussbaum modulation, and high internal command saturation. A hardware-oriented enhanced implementation was then evaluated by preserving the Nussbaum core while adding soft ζ-leakage, low-speed velocity-reference feedforward, and tail-region damping. On the decoupled distal-wrist test bed that this study deliberately adopted as its controlled experimental setting, the best reported 300 s enhanced run reduced the mean absolute error from $10.476^{\circ }$ to $1.054^{\circ }$ and the internal command saturation ratio from 0.450 to 0.012 relative to the direct baseline implementation, with a root-mean-square error of $1.283^{\circ }$ and a 95th-percentile absolute error of $2.145^{\circ }$. The same enhanced implementation was further validated across a multi-amplitude and multi-frequency envelope (sinusoids at $10^{\circ }$ and $40^{\circ }$ for frequencies up to 0.5Hz, plus $15^{\circ }$ and $40^{\circ }$ step responses), and the practical bandwidth limit of the combined low-cost stack was characterised at 1.0–3Hz. The study also showed that Optuna-based real-hardware tuning was useful because it encoded tracking quality, internal command saturation, actuation activity, and adaptation-growth constraints into a reproducible search process.

The main conclusion is therefore limited but practical. NPID control can be transferred to a low-cost current-driven manipulator actuator only when the deployment layer, safety envelope, and adaptation management are treated explicitly. The enhanced implementation reported here should be interpreted as an experimentally validated hardware regularisation under the stated actuator, trajectory, and safety constraints, not as a new general stability result. Future work follows directly from the limitations stated in Section 5: extending the same evidence pipeline to whole-arm, multi-joint trajectory tracking; collecting repeated matched trials for statistical (mean±standard-deviation) reporting; adding a matched classical-PID baseline under the identical current-command deployment layer; testing load variation and no-downtime thermal endurance; and releasing disturbance-rejection datasets that complement the step and sinusoidal evidence reported here.

## Figures and Tables

**Table 1:** Main experimental protocol used in the main real-hardware Nussbaum runs.

Item	Setting
Robot platform	Niryo NED3 Pro
Low-level interface	Direct Dynamixel current command (Mode 0/current-control path)
Primary tested actuator	Dynamixel ID 6/Niryo J5
Dynamixel current scale used for conversion	XM430-W350/XM430 X-series current-register convention, $c_{I}\approx 2.69\;\mathrm{mA}/\mathrm{count}$
Trajectory type	Locked-centre sinusoid
Reference amplitude	$10^{\circ }$
Reference frequency	0.05Hz
Operating centre	$140^{\circ }$ start-centred operating region
Control rate	24 Hz
Main validation duration	300 s
Evidence artifacts	CSV logs, JSON summaries, and publication-style PDF/PNG plots
Core safety envelope	Current clamp, current slew limit, velocity filtering, anti-windup, q-guard, and adaptation freeze on saturation.

**Table 2:** Summary of the Optuna tuning effort used to select the manuscript controller. The 79-trial physical archive is the headline $10^{\circ }$/0.05Hz campaign from which the parameter basin and the final 300s configuration were selected; the additional envelope and step Optuna passes used to retune the later operating points are listed separately.

Archive Component	Trials	Valid	Closed-Loop Time	Best Score
Headline $10^{\circ }$/0.05Hz Optuna archive	79	61	87.9 min	7.129
**Role in selection:** expose a feasible parameter basin under real actuator constraints.				
Envelope and step Optuna passes (supporting)	Several hundred	—	—	—

**Table 3:** Selected Nussbaum run metrics used in the paper. Lower values are better for error and saturation.

Run	MAE (Deg)	RMSE (Deg)	P95 (Deg)	Max (Deg)	Internal Sat. Ratio	Δ*ζ*
Baseline NPID	10.476	15.367	31.709	34.785	0.450	0.729
First enhanced Nussbaum result	1.231	1.513	2.521	8.693	0.054	0.339
Velocity-reference-feedforward baseline	1.156	1.426	2.282	9.081	0.014	0.173
Intermediate refined variant	1.121	1.333	2.202	6.753	0.015	0.208
Final manuscript run	**1.054**	**1.283**	**2.145**	**6.530**	**0.012**	0.194

**Table 4:** Multi-amplitude and multi-frequency real-hardware validation of the enhanced NPID implementation on Dynamixel ID 6.

Amp.	Freq.	MAE (Deg)	RMSE (Deg)	P95 (Deg)	Internal Sat. Ratio	Status
$10^{\circ }$	0.05Hz	**1.054**	**1.283**	**2.145**	**0.012**	Best validated 300s headline run
$10^{\circ }$	0.5Hz	3.63	4.28	7.76	0.655	Bounded and practically usable
$40^{\circ }$	0.05Hz	2.83	3.56	7.04	0.250	Best long-run 300s at $40^{\circ }$
$40^{\circ }$	0.5Hz	8.47	10.61	21.40	0.762	Best short window;

**Table 5:** Real-hardware step-response metrics for the enhanced NPID implementation on Dynamixel ID 6. Both runs start at the $140^{\circ }$ operating centre; the $15^{\circ }$ run targets $125^{\circ }$ and the $40^{\circ }$ run targets $100^{\circ }$. Post-1.5 s and tail metrics are restricted to the post-transient window.

Metric	15° Step	40° Step
Step amplitude (deg)	14.57	39.57
Final measured (deg)	125.07	100.02
Final error (deg)	−0.07	−0.02
MAE (deg)	0.569	0.560
P95 |e| (deg)	1.381	2.778
Max |e| (deg)	6.311	7.565
Post-1.5 s MAE (deg)	0.460	0.502
Post-1.5 s P95 (deg)	0.611	1.323
Tail MAE (deg)	0.171	0.113
Tail P95 (deg)	0.420	0.371

**Table 6:** Final parameter set used for the headline $10^{\circ }$/0.05Hz 300s run. The multi-amplitude/multi-frequency and step runs reported in Table 4 and Table 5 were re-tuned from this basin via additional Optuna passes; the parameters that vary across operating points are max_control, max_current_slew, current_bias_raw, γ/gamma_link, k_delta, qd_lpf_alpha, predict_horizon_sec, tail_kd, and the velocity-reference feedforward parameters stored in the code as fric_coulomb and fric_vel_scale, while the adaptive-core and RBF settings were held constant.

Group	Parameter	Value
Paper core	γ/gamma_link	1.1824623105
Paper core	$k_{\Delta }$/k_delta	0.1336345470
Paper core	$\zeta_{0}$	0.8856929670
Adaptive core	α	7.1072835468
Adaptive core	Γ/gamma_adapt	8.7187500403
Adaptive core	$\sigma_{m}$/sigma_mod	0.2460708825
RBF	Nodes	11
RBF	Width	1.4631904539
RBF	Center range	[−3.2, 3.2]
Hardware-aware extension	$\lambda_{\zeta }$/zeta_leak	0.0237735451
Hardware-aware extension	$\zeta_{\mathrm{soft}}$	0.9891853160
Hardware-aware extension	tail_kd	0.0606430796
Velocity-reference feedforward	$k_{f}$/fric_coulomb	0.0089323247
Velocity-reference feedforward	$v_{f}$/fric_vel_scale	1.2725305725
Deployment	max_control	0.0632
Deployment	$k_{Iu}$/torque_to_current	210
Deployment	command_sign	−1
Deployment	current_bias_raw	−0.1296542137
Safety envelope	max_current_raw	39
Safety envelope	max_current_slew	10.1211443054
Safety envelope	predict_horizon_sec	0.0056165844
Safety envelope	qd_lpf_alpha	0.1383932134
Safety envelope	integral_limit	1.6504936083
Safety envelope	q_abs_guard_deg	35

## Data Availability

The controller source code, run commands, raw closed-loop CSV logs, Optuna trial archives, metric tables, and the figure-generation scripts in the scripts/ directory of the manuscript package (build_main_results_figures.py, build_envelope_figures.py, build_control_architecture_diagram.py) that support the findings of this study are openly available. The project landing page is hosted at https://danielz.co.uk/projects/hardware-aware-nussbaum-pid/ (accessed on 26 June 2026), and the companion GitHub repository (manuscript source, figures, tables, and scripts; accepted-manuscript archive version, 30 June 2026) is at https://github.com/danialza/hardware-aware-nussbaum-pid (accessed on 26 June 2026). Additional archived failure/intermediate evidence and the extra bandwidth-limit probe are provided as Supplementary Materials accompanying this article. Additional bulk data (raw CSV logs and Optuna trial archives) is available from the corresponding author on reasonable request.

## Supplementary Materials

The following supporting information can be downloaded at: https://www.mdpi.com/article/10.3390/s26134212/s1, Figure S1: Supplementary failed and intermediate Nussbaum tests before the final controller; Figure S2: Bandwidth-limit probe at 10°/3 Hz on Dynamixel ID 6, in the same four-panel template as the in-envelope runs; Table S1: Supplementary archive contents, including failed/intermediate runs and bandwidth-limit probe logs.

## Abbreviations

NPID	Nussbaum-PID
MAE	Mean absolute error
P95	95th-percentile absolute error
PID	Proportional–integral–derivative
RBF	Radial basis function
RMSE	Root-mean-square error
TPE	Tree-structured Parzen estimator
