# Supplementary material for: Real-Hardware Deployment of a Nussbaum-Function PID Controller on a Current-Controlled Low-Cost Actuator via Hardware-Aware Optuna Tuning
Source: Sensors (Basel). 2026 Jul 3;26(13):4212. doi: 10.3390/s26134212 (PMC13364380; doi:10.3390/s26134212)
Supplement: Supplementary file 1 [file sensors-26-04212-s001.zip › sensors-4384577-supplementary.pdf]

Supplementary Material

Real-Hardware Deployment of a Nussbaum-Function PID Controller on a Current-Controlled Low-Cost Actuator via Hardware-Aware Optuna Tuning

Danial Zafaranchizadeh Moghaddam, Olga Tveretina, and Abolfazl Zaraki

1 Archived Nussbaum Evidence

The project archive contains additional generated tables and failure-summary figures that document the development path from unsuccessful Nussbaum-PID implementations to the final enhanced real-hardware controller. This supplementary material deliberately avoids reproducing figures already presented in the Results section of the main paper. Instead, Table S1 and Figure S1 preserve the high-error and intermediate tests that explain why the final controller design was necessary.

Table S1: Supplementary failed and intermediate Nussbaum archive items preserved with the manuscript package.

Archive item	Artifact and purpose
Failed/intermediate metric archive	`metrics_comparison_summary.csv`; `fig_failed_test_archive.*`<br>Compact quantitative record of non-final runs with high error, high internal command saturation, or excessive adaptation growth.
Original-law failure	`paper-pure_metrics.*`; `paper-pure_overview.svg`<br>Baseline Nussbaum-PID run with 31.71° P95 error and 45.0% saturation, showing practical loss of control authority near the critical ζ region.
Over-driven slew/current attempt	`bestnow-fric-slewboost-300_metrics.*`<br>Demonstrates that simply increasing actuation aggressiveness can produce 19.80° P95 error and 76.4% saturation.
Early high-spike controller	`stage1-best300_metrics.*`<br>Early 300 s run with usable average tracking but large 8.20° P95 error and 15.45° maximum error.
Slew-focused correction attempt	`tryc-slew16-300_metrics.*`<br>Shows that a narrower slew-focused intervention reduced neither tail spikes nor the long-horizon maximum error sufficiently.
Robust damping attempt	`tryd-robust-300_metrics.*`<br>Additional robust damping worsened tracking to 6.21° P95 error, indicating that damping alone was not the solution.
Bandwidth-limit raw CSVs	`nussbaum_sine_15hz_amp10_optuna_v5_trial0_full.csv`<br>`nussbaum_sine_3hz_sanity.csv`<br>`nussbaum_sine_3hz_struct_best_single.csv`<br>Real-hardware runs released alongside the manuscript that support the bandwidth-limit subsection at 10°/1.5 Hz and 10°/3 Hz.
Step-response raw CSVs	`nussbaum_step15_tailclean_best_single.csv`<br>`nussbaum_step40_best_single.csv`<br>`nussbaum_step15_tailclean_trials.csv`<br>`nussbaum_step_settle120_trials.csv`<br>Real-hardware step runs that support the metrics in the main paper.

The visual supplementary material therefore avoids repeating figures already used in the main Results section. Instead, Figure S1 documents the failed and intermediate tests that motivated the final design choices. These runs are useful scientifically because they show what did *not* work: uncontrolled ζ-growth, over-driven slew/current behavior, large tail errors, and spiky reversal-region behavior before the final controller was selected.

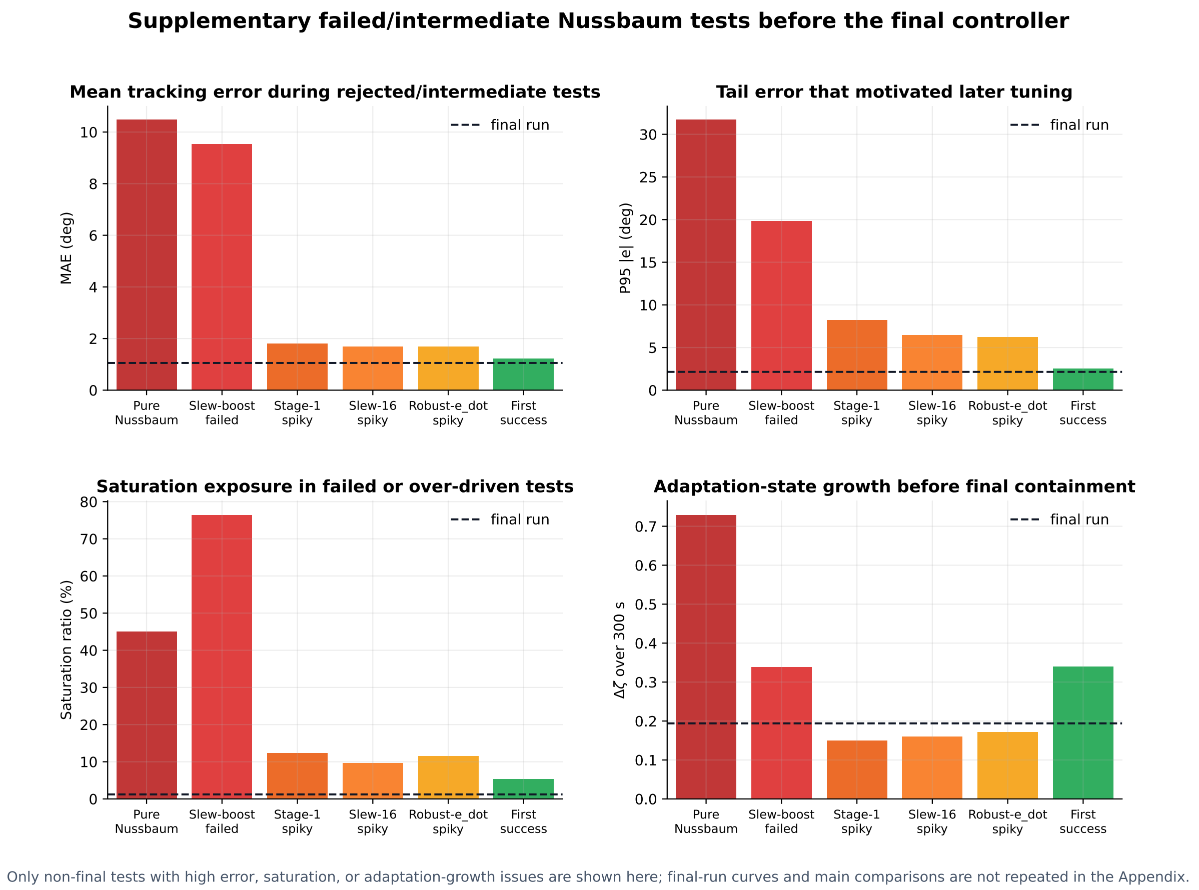

Figure S1: Supplementary failed and intermediate Nussbaum tests before the final controller. The figure intentionally excludes the final-run tracking curves and other main-text comparisons; it only summarizes non-final tests with high error, high internal command saturation, or excessive adaptation-growth behavior. The dashed line marks the final 300 s manuscript run for reference.

2 Additional Bandwidth-Limit Evidence

This supplementary document complements the bandwidth-limit subsection of the main Results section. The main text reports 10°/1.5 Hz as a representative bandwidth-limit probe; the remaining 10°/3 Hz probe is preserved here for completeness because the failure pattern at 3 Hz is qualitatively different from the 1.5 Hz case: the measured-position envelope collapses well below the reference envelope and the joint can no longer realize the demanded motion. The high-amplitude bandwidth probes at 40°/1.0 Hz and 40°/1.5 Hz did not produce a validated tracking-quality candidate within the search budget; their per-trial CSV and JSON logs are released alongside the rest of the supplementary archive (Table S1). These results are consistent with the bandwidth interpretation given in the main text: the practical envelope boundary of the low-cost stack lies near 0.5 Hz, and probes beyond that boundary are reported here as evidence of where the present hardware/software cascade stops being able to deliver phase-faithful tracking, not as failures of the Nussbaum-PID controller itself.

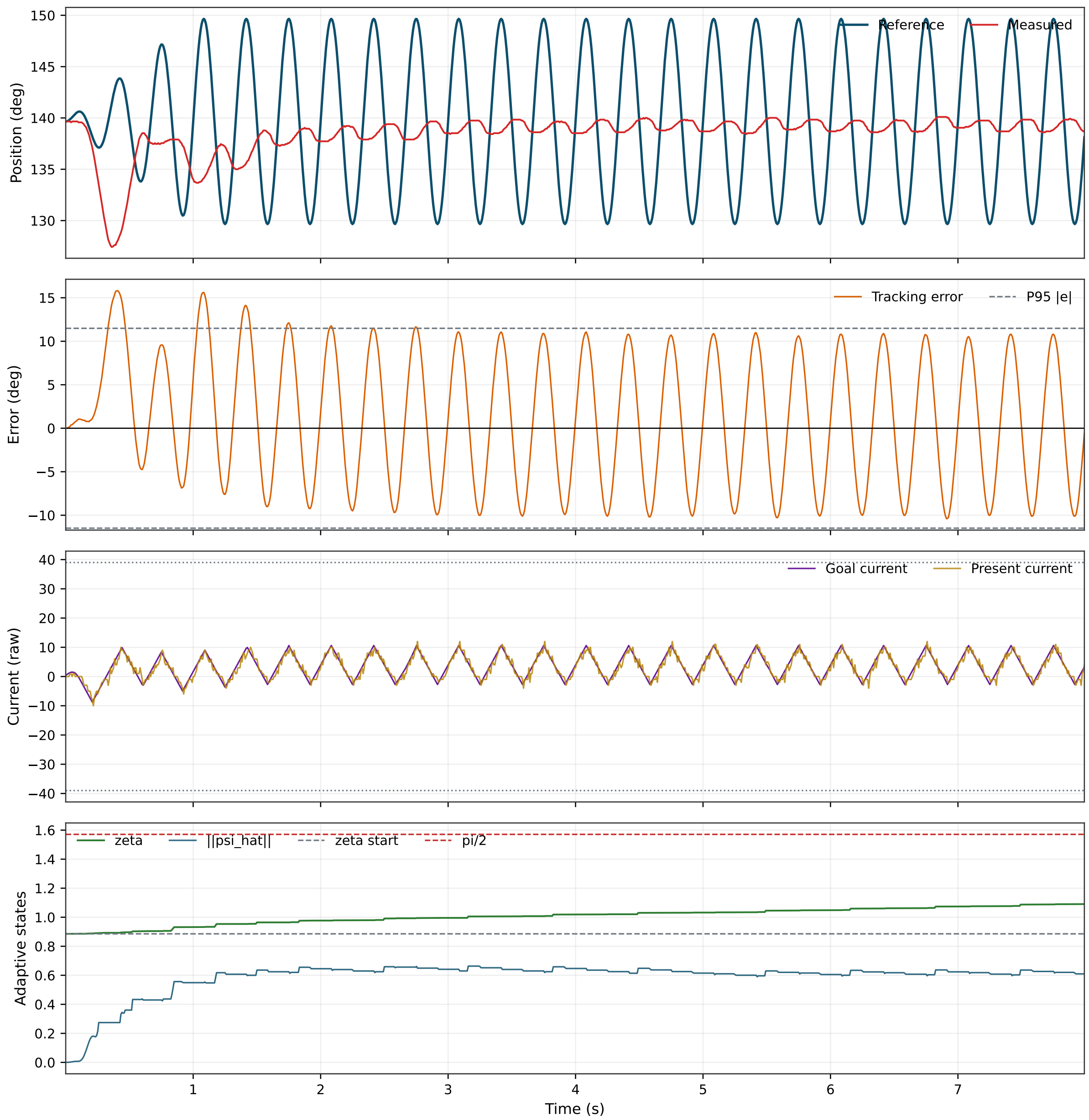

Figure S2: Bandwidth-limit probe at $10^\circ/3\,\mathrm{Hz}$ on Dynamixel ID 6, in the same four-panel template as the in-envelope runs. The measured-position envelope collapses well below the reference envelope, the command u_{pp} sits at the internal `max_control` clamp for the majority of the run, and the adaptive state ζ is held by the leak term while the joint can no longer realize the demanded motion.
